# The Effects of Nanomaterials as Endocrine Disruptors

**DOI:** 10.3390/ijms140816732

**Published:** 2013-08-14

**Authors:** Ivo Iavicoli, Luca Fontana, Veruscka Leso, Antonio Bergamaschi

**Affiliations:** Institute of Public Health, Università Cattolica del Sacro Cuore, Largo Francesco Vito 1, Roma 00168, Italy; E-Mails: lfontana73@yahoo.it (L.F.); veruscka@email.it (V.L.); bergamaschi@rm.unicatt.it (A.B.)

**Keywords:** nanoparticles, endocrine system, endocrine disruptors, health effects, humans, *in vitro* studies, *in vivo* studies, animal, cell lines, invertebrates

## Abstract

In recent years, nanoparticles have been increasingly used in several industrial, consumer and medical applications because of their unique physico-chemical properties. However, *in vitro* and *in vivo* studies have demonstrated that these properties are also closely associated with detrimental health effects. There is a serious lack of information on the potential nanoparticle hazard to human health, particularly on their possible toxic effects on the endocrine system. This topic is of primary importance since the disruption of endocrine functions is associated with severe adverse effects on human health. Consequently, in order to gather information on the hazardous effects of nanoparticles on endocrine organs, we reviewed the data available in the literature regarding the endocrine effects of *in vitro* and *in vivo* exposure to different types of nanoparticles. Our aim was to understand the potential endocrine disrupting risks posed by nanoparticles, to assess their underlying mechanisms of action and identify areas in which further investigation is needed in order to obtain a deeper understanding of the role of nanoparticles as endocrine disruptors. Current data support the notion that different types of nanoparticles are capable of altering the normal and physiological activity of the endocrine system. However, a critical evaluation of these findings suggests the need to interpret these results with caution since information on potential endocrine interactions and the toxicity of nanoparticles is quite limited.

## 1. Introduction

Over the past 50 years, epidemiological data have revealed a significant increase in the incidence and prevalence of a number of adverse effects on human health such as alterations in the development and growth process, disorders of the immune and neurological systems, reduced fertility and the onset of some important diseases such as diabetes, obesity, breast, ovary, testicle and prostate cancer [1–3]. A possible explanation for the increase in these diseases lies in a growing exposure of workers and the general population to contaminants that may exert adverse effects on account of their action as endocrine disrupting chemicals (EDCs). In fact, most studies performed on EDCs have revealed that these compounds may play an important role in the onset of the aforementioned diseases by altering hormonal and homeostatic systems [4–10].

In 2002, the World Health Organization defined EDCs as “an exogenous substance or mixture that alters functions of the endocrine system and consequently causes adverse health effects in an intact organism, or its progeny, or (sub)populations” [11]. The group of chemical substances that belongs to this category is highly heterogeneous: it includes industrial solvents and lubricants and their by-products, dioxins, bisphenol A, polychlorinated biphenyls, persistent organic pollutants (POP), plastic compounds, plasticizers, pesticides such as chlorinated insecticides, imidazoles and triazoles, pharmaceutical agents, chemical compounds that are widely used in cosmetics such as phthalates, and heavy metals such as cadmium, mercury, arsenic, lead, manganese and zinc [1,2,12]. EDCs are found in virtually all regions of the world and typical human exposure in the general population occurs via environmental contamination of the food chain, especially fresh water fish and meat, or through contact with contaminated environmental matrices, while occupational exposure can occur during the production, use and disposal of the aforementioned chemical substances [13,14]. Since exposure of the general population and workers to EDCs is ubiquitous and unavoidable, and the impact on human health due to the known or unknown effects of these chemicals on hormonal systems is great, there is an urgent need to increase efforts to identify the compounds that can behave as endocrine disruptors and to study their molecular mechanisms of action. The Endocrine Disruptor Priority List (EDPL), developed as part of the European Union strategy for EDCs, represents a practical attempt to achieve a wider and more comprehensive knowledge of these substances. It provides a list and categorization (see Table 1) of chemicals that are likely or suspected to be EDCs [15–18].

However, despite the studies carried out in recent years, current knowledge of EDCs is still limited and most research into this issue has concentrated on a few groups of chemical substances such as pesticides or POP, whereas data on a number of other xenobiotics that may act as EDCs is still scant and incomplete [1]. Therefore, to adequately address the issue of EDCs, it is our belief that the first step should be to identify all the possible compounds that can interfere and disrupt the homeostasis and regulatory mechanisms of the endocrine system. This provision is particularly urgent for those chemicals that have recently been used in workplaces and consumer products and whose toxicological profile has not yet been clearly and unequivocally defined. This is the case of nanoparticles (NPs).

The International Organization for Standardization, in ISO/TS 27687, defines a nanoparticle as a nano-object with all three external dimensions in the size range of approximately 1–100 nm [19]. NPs can be subdivided into two main categories, engineered NPs that are intentionally produced with very specific properties or compositions, and incidental NPs, also known as ultrafine particles, that are typically byproducts of processes such as combustion and vaporization and working activities such as welding or grinding [20]. Reducing particle size increases surface area and modifies unique physicochemical properties such as high conductivity, strength, durability, and chemical reactivity [21]. Furthermore, because of their very small size, NPs can enter cells directly by penetrating the cell membrane and may cause interference of important cell functions. The internalization of NPs can occur in a variety of ways and particle size largely influences their endocytic processes and cellular uptake ability. Additionally, several experimental reports have shown that nanoparticle size also affects their internalization in terms of efficiency [22,23]. The industrial applications of NPs are very wide-ranging and include those that may lead to more efficient water purification, stronger and lighter building materials, increased computing power and speed, enhanced generation and conservation of energy, and new tools for the diagnosis and treatment of diseases [24]. In recent years, several *in vitro* studies have assessed the potential adverse health effects of NPs, pointing out their ability to produce reactive oxygen species (ROS), releasetoxic ions, disrupt electron/ion cell membrane transport activity and cause oxidative damage and lipid peroxidation, while the results of *in vivo* studies have shown that these materials can induce adverse effects on the respiratory, cardiovascular and nervous systems [25–27]. However, while nanotechnology and exposure to NPs are growing exponentially, research into their toxicological impact and possible hazard for human health and the environment is still fragmentary and largely incomplete. Consequently, even if nanotechnology and the commercialization of nanoenabled products and devices could help to address serious global problems (energy, transportation, pollution, health, medicine, and food), these benefits may not materialize unless a concerted effort is made to evaluate safety and health concerns regarding NPs.

By summarizing the findings of the *in vitro* and *in vivo* studies that have investigated the effects of NPs on the endocrine system, this review aims to evaluate the potential role of different types of NPs as endocrine disruptors, focusing in particular on their molecular mechanisms of action and on the relationship between their physical and chemical properties and the induction of alterations in endocrine function.

## 2. Impact of Endocrine Disrupting NPs on Reproductive Health

Hormones play a key role in influencing the development of the reproductive system and subsequently in controlling its activities once developed. For this reason, most of the research carried out on EDCs in the last two decades has focused its attention on reproductive health. With regard to the male reproductive system, numerous *in vitro* and *in vivo* study findings have demonstrated that EDCs can exert a number of detrimental effects such as malformed reproductive tissue, poor semen quality (low sperm counts, low ejaculate volume, high number of abnormal sperm, low number of motile sperm), prostate diseases, testicular cancer, and other recognized abnormalities of male reproductive tissues [3,28]. There is also evidence that EDCs may interfere with female reproductive development and function causing adverse effects such as fibrocystic disease of the breast, polycystic ovarian syndrome, endometriosis, uterine fibroids and pelvic inflammatory diseases, breast and reproductive organ tissue cancers and declining sex ratio [3,29].

Recently, the results of studies conducted to assess the potential toxic effects of NPs, have suggested that some of these may pose risks to male and female reproductive health by altering normal testis and ovarian structure, spermatogenesis and sperm quality, oogenesis, follicle maturation and sex hormone levels.

### 2.1. Effects of NPs on the Male Reproductive System

**Figure 1:**
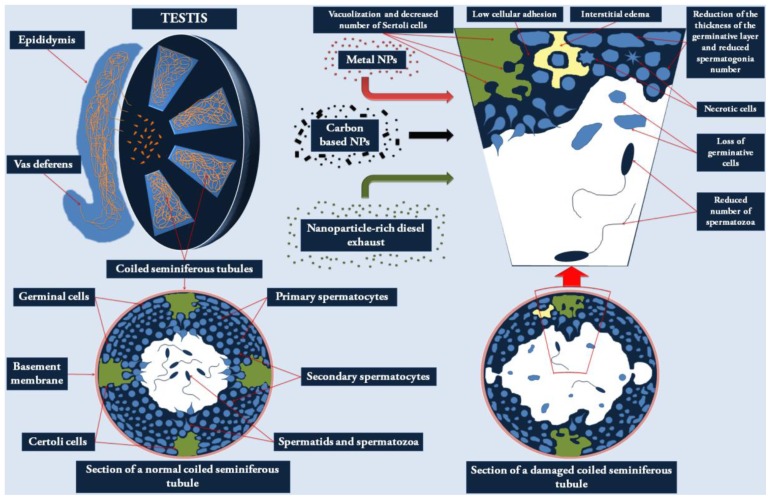
Alterations and impairment of testicular structure induced by exposure to different types of NPs. Metal-based NPs, carbon-based NPs and nanoparticle-rich diesel exhaust (NRDE-NPs) were reported to induce changes in testicular histology of laboratory animals. Metal-based NPs (TiO_2_-NPs) induced the disruption of seminiferous tubules and decreased the number of Sertoli cells; Carbon-based NPs (carbon black-NPs, multi-walled carbon nanotubes) caused vacuolization of seminiferous tubules, seminiferous tubule damage and low cellular adhesion of seminiferous epithelia, partial disappearance or vacuolization of Sertoli cells, reduced thickness of the germinative layer, lower spermatogonia number, vasodilatation and hyperemia; NRDE-NPs induced degenerative and necrotic changes, interstitial edema, desquamation of the seminiferous epithelium, loss of germ cells and spermatozoa.

Most of the adverse effects of NPs on male reproductive function are mainly due to modification of the testicular structure (see Figure 1), impairment of spermatogenesis and alteration in the biosynthetic and catabolic pathways of testosterone (see Table 2).

To investigate damage caused by NPs to testicular constituent cells, three *in vitro* studies evaluated the cytotoxic effects of different types of NPs on mouse Leydig TM3 cells and on the RTG-2 cell line. The exposure of TM3 cells to increasing concentrations (0–1000 μg/mL) of titanium dioxide NPs (TiO_2_-NPs) induced a remarkable and dose-dependent reduction of cell viability [30]. Similarly, in the RTG-2 cell line, exposure to 50 μg/mL (the highest concentration used in this study) of TiO_2_-NPs produced a significant cytotoxicity [31]. In contrast, carbon black NPs (CB-NPs) and diesel exhaust NPs (DE-NPs) caused only slight cytotoxicity in TM3 cells [28], while cerium oxide NPs (CeO_2_-NPs) proved non-cytotoxic on the RTG-2 cell line [32]. With regard to these *in vitro* studies it should be noted that the different degree of observed cytotoxicity may depend on several factors. In fact the effect on the cytotoxicity of cells varies with different NPs and different cells, presumably because of the differing sensibility of cells and the varying sizes, doses, components and physico-chemical properties of the NPs. Furthermore, even when the experimental conditions (cell lines, particle size and concentrations) are quite similar for example in the studies of Vevers *et al*. [31] and Rosenkranz *et al*. [32], the conflicting results could be explained by taking into account the mode of toxicity that may be very different for the various metal oxide NPs, as has been shown for inflammogenic responses [62].

Several *in vivo* studies (see Table 2) have demonstrated that carbon-based NPs, nanoparticle-rich diesel exhaust (NRDE-NPs) and metal-based NPs are able to induce changes in the testicular histology of laboratory animals. These alterations appeared of great importance since they could affect the “micro-environment” required for a correct reproductive function. Intratracheal administration of a 0.1 mg/kg body weight dose of different CB-NPs caused partial vacuolization of the seminiferous tubules in ICR mice [36]. The same research group reported clear seminiferous tubule damage and low cellular adhesion of seminiferous epithelia in the offspring of this animal model after prenatal exposure to 0.2 mg/kg body weight of CB-NPs, demonstrating *in utero* developmental sensitivity to the CB-NP endocrine disrupting insult [38]. Another type of carbon-based NP induced similar damage (reduced thickness of the germinative layer, lower spermatogonia number, partial disappearance or vacuolization of Sertoli cells, vasodilatation and hyperemia) in the testes of BALB/c mice treated with intravenous injections (5 mg/kg/dose) of amine- or carboxylate-functionalized Multi Walled Carbon Nanotubes (MWCNTs) [37]. Severe adverse effects on seminiferous tubules, such as degenerative and necrotic changes, interstitial edema, desquamation of the seminiferous epithelium, and loss of spermatozoa were also observed in Fischer rats exposed via inhalation to different doses (15.37, 36.35 and 168.84 μg/m^3^) of NRDE-NPs [33]. The findings of a similar study, carried out in pregnant rats treated with 148.86 μg/m^3^ of the same NPs, detected the loss of germ cells in the seminiferous tubules of offspring [34]. Since carbon-based NPs of different types and particle sizes, administered to animals by different routes and in a variety of exposure doses caused similar damages, these NPs may share a common mechanism of action that could be the induction of oxidative stress as is suggested by the findings of Bai *et al.* [37] who observed an increase in malondialdehyde levels 15 days after treatment.

With regard to the testicular morphological alterations in offspring, the pre-natal exposure to TiO_2_-NPs of Slc:ICR mice, subcutaneously injected with 100 μg/kg body weight/day, also led to disruption of seminiferous tubules and lower numbers of Sertoli cells in 6 week-old pups [35]. It is worth noting that in this study TiO_2_-NPs were detected in Leydig cells, Sertoli cells and spermatids of the testis of offspring at both 4 days and 6 weeks of age suggesting a direct NP induced insult on the reproductive organ. In contrast, studies carried out by Yoshida *et al.* [38] and Li *et al.* [34] failed to detect NPs in the testis of pups prenatally exposed to CB-NPs and NRDE-NPs, respectively. Consequently, no definitive conclusion can be reached concerning the role NPs may play in causing direct damage to this organ. The damage observed may be related to accumulation of NPs in the placenta, thereby resulting in malnutrition of the foetus. However, the Authors observed no significant differences in body and organ weights between the exposed and control groups in either study.

In contrast with the previous studies, the intravenous administration of 45 and 225 mg/kg ω-methoxy and 45 mg/kg ω-aminoethyl poly(ethylene glycol) capped Au-NPs (mPEG@Au-NP and PEG-NH_2_@Au-NP, respectively) in ICR mice did not cause testicular morphological changes or germ cell apoptosis, even if these NPs had accumulated in mouse testes, passed through the blood-testis barrier, and entered germ cells [39]. Similarly, Morishita *et al*. [40] demonstrated that amorphous silica NPs were able to penetrate the blood-testis barrier and be internalized in cytoplasm and the nuclei of spermatocytes but did not induce any apparent testicular injury in BALB/c mice intravenously injected with 0.8 mg of the aforementioned NPs. Surprisingly, although the total doses of nanomaterials that were administered in these studies were significantly higher than the total dose used in the MWCNT report [37], no testicular morphological alterations were observed. These findings may be due to the different physico-chemical characteristics of the NPs studied, particulalrly their chemical composition, shape and surface functionalizations, since these properties significantly affect the toxicological profile of NPs. Morishita *et al.* [40] have suggested that NP testicular distribution and penetration may depend on the type of material but also on surface modifications as demonstrated by a higher accumulation in the testes of PEG-NH_2_@Au-NP compared to mPEG@Au-NP. Concerning alterations in spermatogenesis (see Table 2), several *in vitro* studies have investigated the potential toxic effects of different types of NPs (mainly metal- and carbon-based NPs) on spermatozoa and their maturation process, principally by evaluating cytotoxicity, DNA damage and modifications in sperm quality parameters. Significant dose-dependent cytotoxicity was observed in the C18-4 spermatogonial stem cell line exposed to concentrations ranging from 0 to 100 μg/mL of silver NPs (Ag-NPs), molybdenum trioxide NPs (MoO_3_-NPs) and aluminum NPs (Al-NPs) [42]. The Ag-NPs were found to be the most toxic. Interestingly, when evaluating the cytotoxicity of several Ag-NPs with different diameters and coatings, the same Authors demonstrated that smaller and hydrocarbon- coated Ag-NPs induced a greater reduction in cell viability [47]. Furthermore, Ag-NPs have been shown to interact with proteins and potentially block signaling by binding to glial cell line-derived neurotrophic factor or its receptor Ret, thereby eliminating ligand receptor interaction, or by interfering with intracellular signaling molecules. The same metal-based NPs and TiO_2_-NPs showed a dose and time dependent cytotoxic effect also in the Ntera2 (NT2, human testicular embryonic carcinoma) cell line and in primary testicular cells obtained from wild type (WT) C57BL6 mice and 8-oxoguanine DNA glycosylase knock-out (Ogg1−/−) (KO) genotype treated with concentrations of NPs ranging from 0 to 100 μg/mL [51]. Similar results were obtained by Terzuoli *et al*. [50] in human spermatozoa treated with different doses (0–500 μM) of Ag-NPs. However, the toxic effects of NPs on germ cells were not confirmed by the studies of Rafeeqi and Kaul and Murugan *et al*. [41,63] who demonstrated a clear biocompatibility between carbon nanotubes (CNTs) and spermatogonial cells and a fullerenol protective effect on the potential oxidative stress damage on spermatozoa, respectively. Once again the findings of these studies demonstrated the importance of different NP physico-chemical properties in determining the toxic effects. In fact, they show the importance of NP size, functionalization and chemical composition in influencing the biologic response of cell systems [41,42,47,63].

With regard to DNA damage, the treatment of mouse epididymal sperm with 0.5 × 10^15^ or 1.0 × 10^15^ particles/mL of gold nanoparticles (Au-NPs) resulted in inhibition of the chromatin decondensation process in gametes [49]. Unfortunately, this adverse effect was not observed by the same research group in a subsequent study conducted on mature gametes [64]. However, a concentration-dependent induction of sperm DNA damage was also observed in human spermatozoa treated with different doses of TiO_2_-NPs (3.73–59.7 μg/mL) and Zinc Oxide nanoparticles (ZnO-NPs) (11.5–93.2 μg/mL) [45]. Conflicting results were obtained for alterations in sperm motility induced by metal-based NPs, a parameter used to evaluate sperm quality. A complete loss of bovine spermatozoa motility was reported after exposure to 2.5 mg/mL of europium oxide NPs (Eu_2_O_3_-NPs) [44], while a 22% loss of motility was detected after treatment with 0.5–50 μM of Au-NPs [48,65]. The latter metal-based NPs also caused a reduction of motility in human fresh semen mixed with 500 μL of Au-NPs and the results of this study showed the ability of NPs to penetrate the heads and tails of spermatozoa inducing fragmentation of the sperm [46]. Finally, in the aforementioned study carried out by Terzuoli *et al*. [50], increasing concentrations of Ag-NPs significantly reduced human sperm motility. Conversely, motility and acrosome reactions of bovine sperm cells were not affected by exposure to 7.35 mM of magnetic iron oxide NPs (Fe_3_O_4_-NPs) coated with poly(vinyl alcohol) [43], or to 2.5 mg/mL of europium hydroxide NPs (EuOH_3_-NPs) conjugated with polyvinyl alcohol or polyvinyl piyrolidone [44]. Interestingly, these results suggest that the toxic potential of NPs may be altered by modifying NP surface functionalization.

The detrimental effects of NPs on the maturation process of male germ cells have also been confirmed by numerous *in vivo* studies. An increase in the testicular apoptotic index and the frequency of round spermatidis with two or more nuclei were observed in CBAB6F1 mice treated via oral gavage with increasing (0–1000 mg/kg) concentrations of TiO_2_-NPs [55]. Similar findings (clear reduction in sperm density and motility) were obtained by Guo *et al*. [52] in ICR mice intraperitoneally injected with 500 mg/kg of TiO_2_-NPs. The intravenous administration to Wistar rats of 5 or 10 mg/kg of Ag-NPs, with different diameters, determined a decrease in epididymal sperm count and an increase in DNA damage in germ cells [56]. In this study, the most severe adverse effects were caused by the smaller particles (20 nm *vs*. 200 nm), suggesting a relevant role of diameter in inducing greater biological reactivity. This finding should be given careful consideration as it may be linked to the different interaction between the NP surface and biological substances present in biological fluids. In fact, NPs may change their physico-chemical characteristics when transferred to body fluids. This may be due to protein corona formed on the NP surface which facilitates the uptake of NPs into cells and cell inner structures, consequently inducing greater toxicity of particles in the nanometer size. Interestingly, the *in utero* exposure of male offspring in pregnant BALB/c mice treated with 50–300 mg/kg of dimercaptosuccinic acid-coated Fe_3_O_4_-NPs also induced a significant reduction in spermatogonia, spermatocytes, spermatids and mature sperm [54]. However, in this study, histological examinations were conducted only on placental tissue and fetus liver, and even if the Fe_3_O_4_-NPs were passed via blood-placenta barrier, it is not clear whether the aforementioned findings were due the direct action of NPs on testes or to an accumulation in the placenta tissue. On the contrary, the administration to ICR mice of 45 and 225 mg/kg of mPEG@Au-NPs and of 45 mg/kg of PEG-NH_2_@Au-NPs failed to result in abnormalities in the sperm collected from the cauda epididymis of the animals [39].

**Figure 2:**
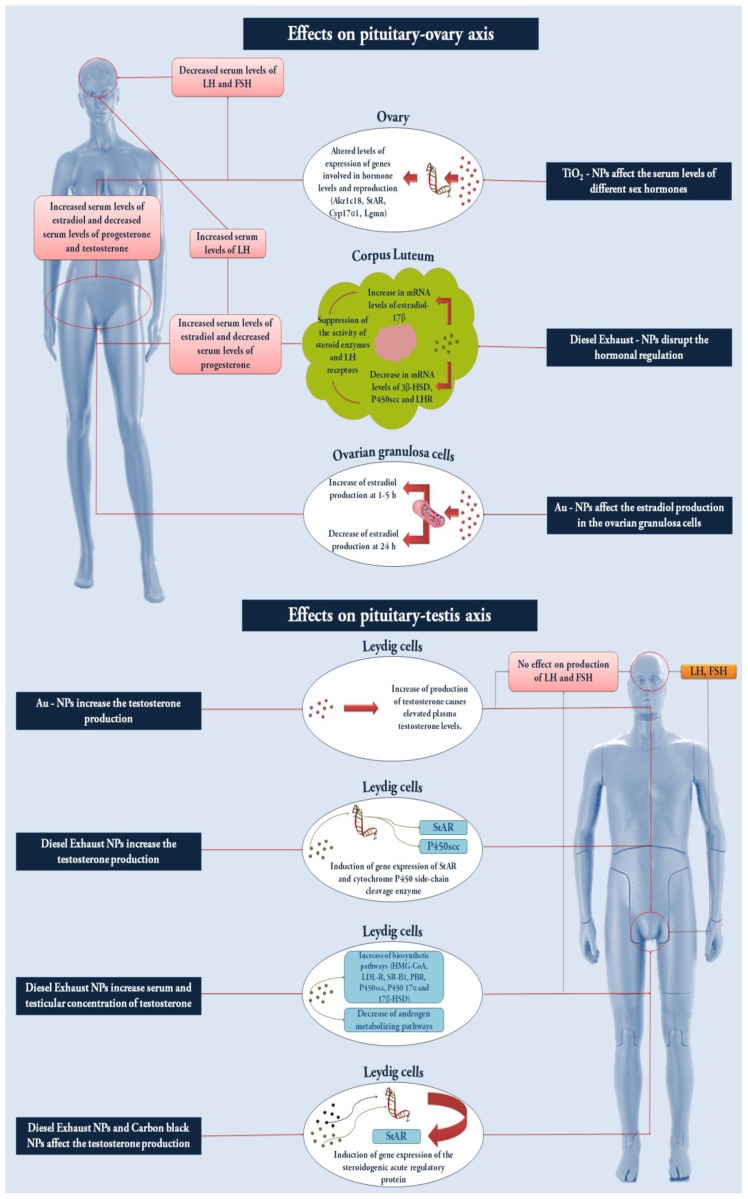
Effects on pituitary-ovary and pituitary-testis axes caused by exposure to different types of NPs. *Effects on pituitary-ovary axis*: Au-NPs induced a greater output of estradiol from ovarian granulosa cells after 1–5 h of treatment, while they decreased the estradiol level after 24 h. TiO2-NP induced changes in hormone levels and ovarian injury in mice may be related to alterations in ovarian gene expression, e.g., Akr1c18, StAr, Cyp17a1, and Lgmn genes were up-regulated. Moreover, TiO2-NPs increased serum levels of estradiol while they decreased those of progesterone, FSH, LH and testosterone in female mice; NRDE-NP exposure led to an increase in LH and estradiol and a reduction in progesterone levels in rats. mRNA expression of the cytochrome P450scc, the 3β-hydroxysteroid dehydrogenase, and the LH receptor were significantly reduced, while that of estradiol-17β was increased by NR-DE exposure, suggesting an impairment in luteal function. NRDE-NP alteration in progesterone secretion was due to the suppression of the steroid enzyme activity and the suppression of the production of LH receptors in corpora lutea; *Effects on pituitary-testis axis*: Au-NPs induced elevated testosterone levels that directly affected the testicular hormone production, since no alterations in LH and FSH plasma values were observed; NRDE-NPs increased the biosynthesis of testicular testosterone. This could be attributed to an increase in the mRNA expression of StAR and cytochrome P450 side-chain cleavage in Leydig cells. Increased levels of serum and testicular testosterone were related to an enhanced expression of genes involved in testicular cholesterol synthesis, such as HMG-CoA, LDL-R, SR-B1, PBR, P450scc, P450 17α, and 17β-HSD induced by NRDE-NPs. Increased testosterone production was due to the impaired balance between androgen-metabolizing- and testosterone biosynthetic-enzymes and not to an LH stimulation, as demonstrated by the absence of alterations in the LH and FSH levels; CB-NPs can affect testosterone production through the induction of StAR gene expression.

Regarding daily sperm production (DSP) and other sperm functional features such as motility and density, adverse effects were reported after both fetal and adulthood exposure to TiO_2_-NPs and CB-NPs. Takeda *et al*. [35] observed a decrease in DSP and epidydimal sperm motility in Slc:ICR mice prenatally exposed to TiO_2_-NPs and the exposure, via inhalation or intratracheal administration, of pregnant C57BL/6J mice to 42 mg/m^3^ of nanosized TiO_2_ or to 67 μg/animal of CB-NPs caused a reduction of DSP in male F1 offspring and lowered sperm production in the F2 offspring, whose fathers were prenatally exposed to CB-NPs [58]. DSP was significantly reduced also in 5 week-old and 10–15 week-old male ICR mice after fetal exposure to 0.2 mg of CB-NPs [38], whereas adult mice subchronically exposed to 0.1 mg of CB-NPs showed a significant reduction in DSP [36]. However, other *in vivo* studies that evaluated alterations in spermatogenesis and sperm quality, using MWCNTs and other carbon-based NPs, failed to confirm the abovementioned findings. In fact, no alterations in the total sperm concentration, motility, percentage of abnormal semen and loss of acrosome integrity were detected in the study conducted by Bai *et al*. [37]. Likewise, Tang *et al*. [57], when evaluating the long term effects of short MWCNTs and polyethylene glycol functionalized s-MWCNTs (PEG-s-MWCNTs) in Kunming mice intravenously treated with a single dose of 100 μg/kg body weight, failed to observe sperm cell toxicity or changes in sperm morphologyFurthermore, in healthy and streptozotocin-induced diabetic male Wistar rats that were administered C_60_ fullerene (C_60_HyFn) at the dose of 4 μg/kg via the oral route, these NPs not only failed to cause significant toxic effects in testicular tissues, but actually increased both sperm motility and epididymal sperm concentration and decreased the abnormal sperm rate, indicating an important anti-oxidant activity [53]. It is worth noting that the lack of effects on the sperm functional features was observed mainly using functionalized and highly pure MWCNTs. Therefore, it can be assumed that the chemical composition and the functionalization of this type of NP play a key role in defining their toxicological profile. For instance, covalent modification of MWCNTs by linking PEG chains to the outside surface of MWCNTs has many advantages because it increases solubility, prolongs circulation half-life *in vivo* and reduces immunogenicity [57]. Another issue involving the capacity of NPs to interfere with the male reproductive system concerns the disruption of normal levels of sex hormones, particularly testosterone (see Table 2 and Figure 2). In fact, the results of two *in vitro* and *ex vivo* studies demonstrated that DE-NPs and CB-NPs are able to affect testosterone production in Leydig cells. In the study carried out by Komatsu *et al*. [30], after 48 h incubation, exposure of the TM3 Leydig cell line to 0–1000 μg/mL of DE-NPs and CB-NPs induced gene expression of the steroidogenic acute regulatory protein (StAR) which is an important molecule in the process of testosterone synthesis, involved as a carrier in cholesterol transfer from the outer to the inner mithocondrial membrane [66]. Moreover, the evaluation of testosterone production in interstitial testicular cells, dissected from male C57BL/Jcl mice exposed to 152.01 μg/m^3^ of NRDE-NPs, showed that this parameter significantly increased both with and without human chorionic gonadotropin stimulus [59].

Alterations in testosterone levels leading to impaired male reproductive function have also been investigated in numerous *in vivo* studies. Li *et al*. [33] observed that, in Fischer 344 rats, inhalation exposure to low (15.4 ± 1.0 μg/m^3^) and medium (36.4 ± 1.2 μg/m^3^) concentrations of NRDE-NPs significantly increased plasma and testicular testosterone, whereas this did not occur with exposure to a high concentration (168.8 ± 2.7 μg/m^3^), thereby clearly indicating an inverted-U dose-response. However, this phenomenon should be carefully evaluated since it may be due to agglomeration of NPs at the highest dose that reduces the activity of the nanoform. Plasma luteinizing hormone (LH) and follicle stimulating hormone (FSH) concentrations did not change significantly, while increased concentrations of the plasma and testicular immunoreactive (ir-) inhibin, a protein complex which inhibits FSH synthesis and secretion were observed in exposed groups. These findings suggest that the increased levels of testosterone were not related to LH stimulation but were due to the disrupted balance between androgen-metabolizing- and testosterone biosynthetic-enzymes. When attempting to clarify the mechanism responsible for the rise in testosterone levels, the same research group [60] confirmed results obtained by Komatsu *et al*. [30], thus proving that increased testosterone biosynthesis could be attributed to an increase in the mRNA expression of StAR and cytochrome P450 side-chain cleavage (P450scc), an enzyme responsible for the conversion of transported cholesterol to pregnenolone in Leydig cells [67]. Similar findings and action mechanism were obtained in the same animal model exposed to high (149 ± 8 μg/m^3^) and low (38 ± 3 μg/m^3^) NRDE-NPs concentrations [61]. Furthermore, higher levels of testosterone were also observed in male C57BL/Jcl mice treated with 152.01 μg/m^3^ of NRDE-NPs and the increase in this hormone was related to an enhanced expression of genes involved in testicular cholesterol synthesis, such as HMG-CoA, LDL-R, SR-B1, PBR, P450scc, P450 17α, and 17β-HSD [59]. Interestingly, in pregnant Fischer F344 rats, exposure to 148.86 μg/m^3^ of NRDE-NPs caused changes in offspring hormone levels involving decreased FSH and testosterone and increased ir-inhibin concentrations that were quite different from those observed in adult animals [34]. The possibility of NPs affecting reproductive function in different ways depending on the period of exposure *i.e.*, during adulthood or the fetal period, is also corroborated by the findings of Yoshida [36,38] who reported elevated testosterone levels in ICR adult mice treated with CB-NPs but no significant increase in the offspring after in utero exposure. Elevated testosterone levels were also detected following the administration of PEG-NH2@Au-NP in ICR mice, while no alterations in LH and FSH plasma values were observed [39]. Taken together, these results suggested that modified-Au-NPs may act as endocrine disruptors that have a direct influence on testicular hormonal production, since pituitary LH and FSH levels remained unmodified. Moreover, NP surface chemistry would seem to play an important role in inducing hormonal alterations, in fact, unlike PEG-NH2@Au-NP, the mPEG@Au-NPs did not cause an increase in testosterone concentration. As confirmation of the importance of the functionalization of NPs in affecting sex hormone levels, no alterations in testosterone, LH and FSH plasma levels were observed in BALB/c mice, subacutely exposed to functionalized MWCNTs [37].

The aformentioned studies that investigated the adverse effects of NPs on male reproductive function used different types of NPs, cell lines, exposure routes and doses. Consequently, caution is needed when interpreting their findings, and reaching a definite conclusion poses a considerable challenge. Any evaluation of these results may take into account the fact that the exposure concentrations used in most of the studies were very high (up to 2.5 mg/mL and 1000 mg/kg for *in vitro* and *in vivo* studies respectively) and would probably be difficult to find in an environmental exposure context. Finally, it should be noted that the different toxic effects induced by NPs were strongly related to their physico-chemical properties which, in most of the studies we reviewed, were not adequately analyzed and defined. In fact, NP characterization often focuses only on the chemical composition and particle size (see Table 2) while, in our opinion it is extremely important to assess other relevant parameters such as surface area, reactivity and functionalization, charge, solubility, degree of agglomeration and number concentration.

### 2.2. Effects of NPs on the Female Reproductive System

In recent years, numerous *in vitro* and *in vivo* studies have evaluated the potential adverse effects of NP exposure on the female reproductive system. Study findings have demonstrated that several NPs can interfere with normal female reproductive function by inducing cytotoxic effects on ovarian structural cells, impairing oogenesis and follicle maturation, and altering normal sex hormone levels (see Table 3).

Different types of NPs, in particular TiO_2_-NPs, carbon-based NPs and silica NPs, were studied in order to define their cytotoxic potential on Chinese hamster ovary cells (CHO), epithelial-like ovarian constituent cells. With regard to metal-based NPs, data currently available have shown the ability of TiO_2_-NPs to be internalised by CHO-K1 and to induce a dose-dependent decrease in cell viability following both acute [68,71,72] and subacute exposure [76,77]. Anatase TiO_2_-NPs reduced cell viability in relation to the increasing dose applied (0–100 μg/mL) and the increasing ROS concentrations induced [68]. Similarly, anatase and rutile TiO_2_ NPs caused a cytotoxic effect in CHO cells treated with concentrations ranging from 25 to 325 μg/mL [71]. The results of this study showed that particle size and crystal phase composition can significantly affect the cytotoxic behaviour of NPs since 10–20 nm anatase yielded the highest level of cytotoxicity followed by 50–60 nm anatase and 50–60 nm rutile. Comparable results were obtained by Di Virgilio *et al*. [72] on treating CHO-K1 cells with 0–100 μg/mL of non-characterized (in terms of crystal phase composition) TiO_2_-NPs. Moreover, cell viability was negatively affected by acute and subacute exposure to concentrations of >50 μg/mL of anatase TiO_2_-NPs [76,77]. The most significant toxic effects were observed following treatment with particularly high doses of TiO_2_-NPs (100 and 200 μg/mL). However, these results should be interpreted with caution since the applied doses may not be representative of exposure levels in the general population or in occupationally exposed subjects. Aluminium oxide NPs (Al_2_O_3_-NPs), another type of metal-based NP, were able to penetrate the cytoplasm of CHO-K1 cells treated with 0–100 μg/mL [72], inducing dose-dependent cytotoxic damage. However, this was not so severe as the damage caused by TiO_2_-NPs and began at higher concentrations suggesting that the chemical composition of NPs is also important in causing toxic effects.

In a study on carbon-based NPs that examined the effects on CHO cell viability, of silicon carbide nanowires (SiCNWs) obtained from the reaction of MWCNTs with silicon monoxide, Jiang *et al*. [73] reported an important decrease in the reproduction rate. The main mechanism leading to SiCNW cellular toxicity was identified in the induction of the mitogen-activated protein kinase cellular signaling pathway and in the over expression of cyclossigenase-2. In the same cell line, transfected with the macrophage receptor with collagenous structure (MARCO), treatment with increasing concentrations (1–100 μg/mL) of MWCNTs caused significant cytotoxicity that was probably related to the incomplete inclusion of NPs by the cell membrane [79]. Furthermore, treatment of female granulosa cells, collected from infertile women, with calcium phosphate-NPs (10 and 100 μM), caused an accumulation in the S phase of the cell cycle with an increased apoptotic rate [74]. With regard to cytotoxicity in ovarian cells it is interesting to note that metal-based and carbon-based NPs may produce an effect through different molecular mechanisms of action such as the induction of oxidative stress and the activation of MAPKs cellular signaling/over-expression of COX-2, respectively.

In contrast, when CHO cells were seeded on an aligned MWCNT substrate, no toxicity was observed: the cells grew well and were aligned, and only a few cells elongated along the axis of the MWCNT bundles [75]. Studies have also demonstrated good biocompatibility of mesoporous silica NPs with human ovary cells. In fact, when naked mesoporous silica NPs were applied to ovarian NIH-OVCAR3 epithelial cancer cells (30, 75 μg/mL), no altered cell morphology, metabolism or cell loss was reported, although there was evidence of internalization of the NPs [78]. Comparable results were obtained in NIH-OVCAR3 and SKOV3 cancer cells treated with 20 μg/mL of greater or differently functionalized (carboxyl or amine modified) mesoporous silica NPs. In the same study, carboxyl-modified polystyrene-NPs were not toxic for NIH-OVCAR3 and SKOV3 cells at a concentration of 75 μg/mL, while polystyrene NPs functionalized with amine groups showed significant cytotoxic effects [78]. The absence of toxic effects of silica NPs in the latter study, compared to those previously analyzed, may be related to a number of factors, including the different type of NPs used (mesoporous silica NPs vs metal and carbon-based NPs), cell models adopted (NIH-OVCAR3 and SKOV3 vs CHO) and lower exposure concentrations, whereas the different functionalization of polystyrene-NPs seems to play a decisive role in the induction of cytotoxic effects.

Conflicting results were obtained for the genotoxic effects of metal-based NPs on the CHO cell line. In fact, at doses < 5000 μg/mL, anatase and rutile TiO_2_-NPs failed to produce any increase in chromosomal aberration frequencies in CHO-WBL cells, either in the absence or presence of UV [69]. Similarly, no structural or numerical chromosome aberrations were observed in CHO cells exposed to several doses (25–2500 μg/mL) of rutile TiO_2_-NPs [70] and no genotoxic effects were detected in the CHO-K1 cell line treated with increasing concentrations (0, 10, 20, or 40 μg/mL) of anatase TiO_2_-NPs [76]. Conversely, studies carried out by Zhu *et al*. [71] and Di Virgilio *et al*. [72] reported an increased frequency of sister chromatid exchange, micronuclei and DNA strand breaks. Moreover, Al_2_O_3_-NPs induced a dose-dependent response of micronuclei frequency in the whole dose-range studied [72]. Comparable results were obtained with SiCNW exposure [73]. The different experimental design as well as the chemical composition of the NPs and the crystal form of the TiO_2_-NPs were probably responsible for most of the inconsistencies observed. In particular, it is worth noting that 3 h [69] and 4 h [70] exposure periods may not be long enough to detect genotoxic effects, indicating that the response is also dependent on the exposure time.

Oocyte viability and maturation are extremely sensitive to microenvironment changes and extracellular chemical compounds [81]. Consequently, several *in vitro* studies have been carried out to assess the potential adverse effects of NPs on female germ cells (see Table 3). The findings of these studies, which indicated an alteration in oocyte maturation and fertilization and their subsequent embryonic development, point to the role of TiO_2_-NPs and Quantum Dots (QDs) as endocrine disruptors. In fact, Hou *et al*. [80] demonstrated that TiO_2_-NPs (12.5–50 μg/mL) inhibited rat follicle development and oocyte maturation, with a dose-dependent reduction in the survival rate of follicles, formation rate of antral follicles and release rate of cumulus-oocyte cell complexes. Similar results, (dose-dependent decrease in oocyte maturation rate, reduced fertilization, impairment of cell proliferation and dose-dependent enhanced blastocyst apoptotic rate) were observed on oocytes isolated from female ICR mice exposed to 0–500 nM of cadmium selenium core QDs (CdSe-core-QDs). However, the coating of QDs with a zinc sulfide shell prevented the cytotoxic effects on oocytes, thereby suggesting that QD surface chemistry plays an important role [81]. The results of this study are particularly intriguing since they point out the importance of surface functionalization of QDs in altering their biological effects. In fact, the Authors suggested that the ZnS coating of CdSe QDs prevented cytotoxicity by blocking surface oxidation and the subsequent release of Cd^2+^ ions, mechanisms that are correlated with the induction of cell death by CdSe-core QDs. Therefore, it is plausible to assume that NPs may exert their toxic effects by dissolving to become a source/reservoir of toxic cations. The possible release of ions from metal-based NPs and QDs is an important topic that needs careful consideration when evaluating the potential toxic effects of these NPs. The same research group investigated the effects of CdTe/ZnTe QD-Transferrin (QD-Tf) bioconjugates on follicle development and oocyte maturation in female Kunmimg mice. Their findings revealed a dose-dependent up-take of QDs-Tf by theca and granulosa cells and a significant decrease in the rate of antrum cavity formation and of the ratio of oocytes to first polar body [84]. These results suggested that the high uptake of QDs-Tf could potentially disturb oocytes to form antrum cavity and advance in maturation, as previously reported by Xu *et al*. [82] and Wang *et al*. [83] who, after treating Kunming mice immatured oocytes with CdSe/CdS/ZnS QDs, observed a significant reduction in the oocyte maturation process. The high uptake of QDs-Tf was probably due to the small hydrodynamic diameter and the bioconjugation of QDs that can facilitate penetration and increase impact on oocytes.

Impairment of the female reproductive function, due to some metal-based NPs and QDs that modify oogenesis and follicle maturation, has also been observed in several *in vivo* studies (see Table 3). Wang *et al*. [85] reported that administration of TiO_2_-NPs to zebrafish *Danio rerio* at concentrations of 0.1 and 1.0 mg/L, skewed the distribution of follicular developmental stages toward immature statuses and reduced the expression of gene coding for growth factors implicated as paracrine stimuli in oocyte maturation. Interestingly, when zebrafish *Danio rerio* were exposed to the same NP concentrations for a shorter period of time, female gonads showed a normal spread of oocyte development stages in all exposed groups [88]. However, the group treated with the highest TiO_2_-NPs dose (1.0 mg/L) yielded a much lower production of eggs and viable embryos. The differences in oocyte development stages observed in these studies could be due to the different exposure periods (13 weeks *vs*. 14 days). Accumulation of anatase TiO_2_-NPs in the ovary tissues of CD-1 (ICR) female mice, intragastrically treated with 10 mg/kg of these NPs, caused atresia of primary and secondary follicle development and led to increased Ca, Na, K, and Zn contents, and decreased Mg, Cu, and Fe contents [86]. Modification of the equilibrium of these elements in the ovaries might adversely affect their normal physiology and consequently lead to alterations in the female germ cell maturation process. Gene expression analysis in ovary tissues of adult sheepshead minnows (*Cyprinodon Variegatus*) exposed to 10 μg/L of Ag-NPs, revealed a dramatic transcriptional response indicating that exposure to these NPs can cause reproductive dysfunction in estuarine fish, even though the absence of ovary morphological and developmental alterations did not support this hypothesis [87].

It has been demonstrated that a number of NPs are able to alter physiological sex hormone levels in the female as well as the male reproductive system (see Table 3 and Figure 2). In *in vitro* studies, Au-NPs and QDs were found to alter estrogenic hormonal levels. The exposure of rat ovarian granulosa cells to 2.85 × 10^10^ NPs/mL of metal-based NPs induced a greater output of estradiol after 1–5 h of treatment, while a significant decrease was observed after 24 h. Initial estradiol accumulation could be ascribed to alterations induced by the internalized Au-NPs in the cleavage activity of cholesterol into pregnenolone, and in mitochondrial permeability, while the subsequent inhibition of estradiol production was probably due to impairment of the mitochondrial steroidogenic machinery [89]. The same mechanism of action has been proposed to explain the increase, at the 4th–8th day post-treatment, in 17 β-estradiol secretion into granulosa cells treated with the highest QD-Tf concentration. 17 β-estradiol secretion was less than in controls at lower QD-Tf concentrations [84]. Unlike previous studies, the production of progesterone and estradiol in the granulosa cells was not affected by treatment with calcium phosphate-NPs and no alterations were observed in the levels and expression of mRNA encoding P450scc, P450arom, and StAR [74]. These results did not agree with the findings of Stelzer and Hutz [89] and Xu *et al.* [84] and it is possible that these disparities may be due to differences in dosage, chemicals, particle size, and cell model in the aforementioned studies.

The issue concerning alterations in sex hormone levels caused by exposure to metal-based NPs and NRDE-NPs, has also been addressed by some *in vivo* studies which have, however, provided conflicting results. In fact, serum levels of estradiol significantly increased, while progesterone, FSH, LH and testosterone levels diminished in female CD-1 (ICR) mice sub-chronically treated with TiO_2_-NPs [86]. Nevertheless, the administration of zinc oxide NPs (ZnO-NPs) to female Wistar rats at a concentration of 333.33 mg/kg did not alter serum sex hormone levels such as FSH, LH and estradiol [91]. Once again the conflicting results observed in these two studies may be related to several factors such as the different type and size of NPs, and the varying doses of exposure and length of treatment. With regard to the latter parameter, it is worth noting that a subchronic intragastric exposure (90 days) [86] was able to induce a significant increase in estradiol levels, whereas acute administration via the same route [91] failed to determine hormonal alterations. This highlights the need, when extrapolating results from *in vivo* studies, to carefully evaluate also the periods of time used for an investigation. Finally, with regard to NRDE-NPs, exposure of pregnant Fischer 344 rats to 148.86 μg/m^3^ of these NPs led to an increase in LH and a reduction in progesterone levels, whereas no significant differences were observed in FSH, prolactin, ir-inhibin and testosterone levels [90].

As observed for the male reproductive system the effects reported in these studies are largely dependent on many factors related to the experimental design and the physico-chemical characteristics of the NPs investigated. Evaluation of these results should therefore take these variables into account. Moreover, when analysing the endocrine effects on the female reproductive system, it should be noted that the exposure concentrations used in both *in vitro* and *in vivo* studies were very high, up to 5 mg/L and 333.33 mg/kg, respectively.

### 2.3. Estrogenic Effects of NPs

Several *in vivo* studies have investigated the potential estrogenic effects of different types of NPs in male and female organisms (see Table 4). However, their findings are limited, fragmentary and often conflicting, especially with regard to the adverse effects on male organisms.

The estrogenic effects of Ag-NPs and QDs were investigated in aquatic species through the analysis of two proteins: vitellogenin (VTG), the yolk-precursor lipoprotein, and choriogenin (Chg), the precursor protein of the inner layer subunits egg envelope (chorion) of the teleosts which are under estrogenic control and consequently have a role as a biomarker of estrogens and endocrine disruption in oviparous organisms. In this context, Pham *et al*. [92] observed a conspicuous mRNA induction of VTG-1 and Chg-L in the livers of male Medaka (*Oryzias latipes*) fish exposed to Ag-NPs at concentrations of 1 and 25 μg/L. On the contrary, exposure of sexually immature rainbow trout (*Onchorynchus mykiss*) to concentrations of Ag-NPs ranging from 0 to 6 μg/L induced a significant reduction in liver expression of VTG-like proteins [93]. Similarly, studies to investigate the estrogenic effects of QDs also provided conflicting results since the administration of (CdS)/CdTe capped-QDs at doses of 1, 2 and 6 μg/L induced a clear up-regulation of VTG and a down-regulation of its receptor in juvenile rainbow trout (*Oncorhynchus mykiss*) [94], while the exposure of male sticklebacks (*Gasterosteus aculeatus*) to 0–500 μg/L of CdS-QDs failed to show any alteration in VTG expression [95]. With regard to the effects caused by Ag-NPs the different induction of VTG could be due to the variation in exposure doses used in the studies of Pham *et al*. [92] and Gagnè *et al.* [93] (1, 25 μg/L *vs*. 0.06, 0.6 and 6 μg/L, respectively). Neverthless, in the QD studies the up-regulation of VTG was evident at the lowest exposure concentrations (1, 2 and 6 μg/L *vs*. 0–500 μg/L) thus suggesting that the effects may be correlated to other aspects such as the different functionalization of QDs. However, an assessment of these estrogenic effects should take into consideration differencies in species and sexual maturation.

Likewise, the evaluation of progesterone production in male laboratory animals exposed to NRDE-NPs did not provide consistent results. In fact, Li *et al*. [96] demonstrated a lower basal release of progesterone and corticosterone from adrenal cells obtained from male C57BL/Jcl mice exposed to 152.01 μg/m^3^ of NRDE-NPs, while greater concentrations were determined in the low-dose (41.73 μg/m^3^) NRDE-NP treated group compared to controls. However, the same research group observed increased plasma progesterone levels in male F344 rats exposed to middle (36.35 μg/m^3^) or high (168.84 μg/m^3^) NRDE-NP concentrations and decreased hormone levels in rats administered with low (15.37 μg/m^3^) or high (168.84 μg/m^3^) NRDE-NP doses after 4 and 8 weeks treatment, respectively [33]. Furthermore, immature male F344 rats prenatally exposed to the same NPs (148.86 μg/m^3^ to pregnant mice) showed significantly reduced serum progesterone concentrations [34]. Finally, a study on the estrogenic effects of carbon-based NPs reported that treatment of male zebrafish (*Danio rerio*) with C_60_ fullerene reduced the biodisponibility of the synthetic estrogen, 17α-ethinylestradiol [97,98].

In female organisms, the estrogenic effects of QDs and CB-NPs appear to be more evident. In fact Jain *et al*. [99] demonstrated the metalloestrogenic effects of CdTe-QDs on human breast cancer MCF-7 cells. These NPs were found to induce estrogen-related genomic and non-genomic signaling pathways and increase estrogen receptor-α activation and biphasic phosphorylation of AKT and ERK1/2, similar to 17β-estradiol action [99]. In the same study, the treatment of BALB/C prepubescent and virgin ovariectomized female mice with CdTe-QDs caused a two to three fold increase in uterine weight, comparable or greater than that induced by 17β-estradiol. Interestingly, these results suggest that Cd^2+^ released by CdTe-QDs is able to bind to the estrogenic receptor (ER). Exposure of C57BL/6 mice to CB-NPs at a total dose of 11, 54 and 268 μg/kg body weight during gestation, induced a significantly earlier onset of puberty in the offspring, assessed as time of vaginal opening [100].

## 3. Impact of Endocrine Disrupting NPs on Thyroid Function

Thyroid hormones are involved in numerous physiological processes as regulators of metabolism, bone remodeling, cardiac function and mental status. Moreover, thyroid hormones are of special importance in fetal development, particularly in brain development, since the absence or alteration of their physiological levels reduces neuronal growth and differentiation in the cerebral cortex, hippocampus, and cerebellum [101,102]. In recent years, epidemiological studies have indicated that even minor changes in thyroid homeostasis may affect fetal neurological development [103]. Since exposure to thyroid EDCs may significantly affect public health, and studies on most chemicals with thyroid-disrupting potential have been sporadic and have failed to provide consistent results [104], there is clearly a need to evaluate the possible action of endocrine disrupting NPs on thyroid function.

To date, in both *in vitro* and *in vivo* models, conflicting results have been reported regarding the role that different metal-based NPs and QDs play in influencing thyroid hormonal pathways (see Table 5). Hinther *et al*. [105] investigated the effects of exposure to Ag-NPs, ZnO-NPs and QDs on thyroid hormone (TH) signalling in frog tissue using a cultured tail fin biopsy assay derived from Rana catesbeiana tadpoles. Results showed that exposure to 5–10 nM of Ag-NPs and to 0.1 nM of QDs alone induced a reduction in levels of transcripts encoding the TH-induced receptor β (TRβ) and TH-repressed Rana larval keratin type I (RLKI), whereas treatment with ZnO-NPs had no effect on TRβ and RLKI transcript levels. Furthermore, Ag-NPs were able to affect TH-action on TRβ and RLKI transcripts according to a nonmonotonic “inverted-U” hormesis response that is characteristic of endocrine disruption. In contrast, the administration of chromium NPs (Cr-NPs) to heat-stressed Sprague-Dawley rats did not bring about significant modifications in thyrotropic-stimulating hormone (TSH), free triiodothyronine (FT_3_) and free thyroxine (FT_4_) serum levels, indicating that these NPs do not affect the metabolism of thyroid hormones [106].

Clearly, current data concerning the impact of endocrine disrupting NPs on thyroid function are conflicting and very limited. Moreover, since and adequate comparison of findings cannot be made on account of differences in experimental conditions, it is not possible to evaluate the overall effects of NPs on thyroid.

## 4. Impact of Endocrine Disrupting NPs on Insulin Action and Metabolism

The findings of several toxicology and epidemiology studies have suggested that various EDCs are involved in an increasing number of metabolic disorders, including insulin resistance (IR) and IR-related co morbidities, such as obesity, type 2 diabetes mellitus (T2DM) and polycystic ovary syndrome [12,107,108]. These particular EDCs are also known as metabolic disruptors [109]. Recently, a number of *in vitro* and *in vivo* studies have evaluated possible metabolic disruption of insulin-signalling pathways and insulin production caused by exposure to different metal-based NPs (see Table 5).

In this context, Gurevitch *et al*. [110] investigated the potential role of TiO_2_-NPs in the etiology of metabolic/endocrine disorders such as obesity and closely- related IR. In this study, the exposure of Fao rat hepatoma cells to 50 and 200 μg/mL of TiO_2_-NPs impaired insulin response and induced insulin resistance through direct interference with insulin-signalling pathways and indirect inflammatory activation of the macrophages. In another *in vitro* study, an increase in cell viability, ATP/ADP ratio and secretion of insulin in response to glucose stimuli was observed in isolated pancreatic islets treated with CeO_2_-NPs at a concentration of 100 nmol/L, either alone or in combination with 30 nmol/L sodium selenite [111].These findings could possibly be ascribed to the proinflammatory action of TiO_2_-NPs [110] and to the anti-oxidant potential of CeO_2_-NPs [111] which may exert a different effect on the insulin cascade and release, respectively.

In contrast, dietary addition of 300 and 450 μg/kg of Cr-NPs caused a significant decrease in serum insulin levels in Sprague-Dawley rats [113]. Similarly, lower concentrations of insulin and cortisol and higher levels of insulin growth-factor (IGF)-1 were detected in the same animal model treated with a basal diet supplemented with 150–450 μg/kg of Cr-NPs [106]. Comparable results were obtained by Wang *et al*. [112] when administering 200 μg/kg of Cr-NPs to crossbred pigs, while an increase in serum levels of IGF-1 was reported in Swiss mice intranasally instilled with 1.5 mg/kg of double walled-CNTs [114]. The reduction in peripheral insulin levels may have been due to an enhanced binding of insulin to its receptors on targeted tissues such as muscle and fat since Cr is thought to facilitate the interactions between insulin and insulin receptors and to promote hormone internalization into cells [125]. The same Authors provided confirmation of the protective effects of CeO_2_-NPs on pancreatic islets in streptozotocin induced diabetic Wistar rats intraperitoneally injected with these NPs (60 mg/kg) alone, or in combination with sodium selenite (5 μmol/kg/day). The findings of this study showed a significant improvement in diabetes biomarkers including oxidative stress, energy compensation (ADP/ATP), and the glycemic and lipid profile [115].

## 5. Impact of Endocrine Disrupting NPs on the Neuroendocrine System

Chromaffin cells are neuroendocrine cells found mainly in the medulla of the adrenal gland and in other ganglia of the sympathetic nervous system. In these cells the release of numerous catecholamines into the blood e.g., adrenaline, noradrenaline, enkephalin and a range of biologically active peptides occurs through multiple intracellular (chromaffin cell excitability, Ca^2+^ signaling, exocytosis, endocytosis) and intercellular pathways (splanchnic nerve-mediated synaptic transmission, paracrine and endocrine communication, gap junctional coupling) in response to a wide variety of stress-related signals [126,127]. Chronic hypoxia is also included among these signals and can significantly affect catecholamine secretion by increasing the number of secreted vesicles at low voltages, although it does not alter the mechanism of catecholamine release [128]. Catecholamine secretion usually takes place in situations of fear, anxiety or organic stress. However, the release of hormones from adrenomedullary chromaffin cells can also be triggered by a number of chemicals that reach the adrenal medulla via the bloodstream [129]. For this reason, in recent years several *in vitro* studies have been performed in order to verify the endocrine disrupting activity of NPs (especially metal-based NPs), on chromaffin cells (see Table 5). Study findings have shown that NPs have the potential to disrupt the physiological function of neuroendocrine cells by altering their catecholamine levels or compromising the exocytotic machinery.

Hussain *et al*. [117] observed a dose-dependent depletion of dopamine (DA) and dihydroxyphenylacetic acid (DOPAC) in PC-12 cells exposed to 1–100 μg/mL of manganese oxide NPs (MnO-NPs). In the same study, treatment with Ag-NPs induced similar results but only at a concentration of 50 μg/mL, while homovanillic acid (HVA) levels decreased at 50 μg/mL in both MnO-NP and Ag-NP exposure. Comparable results were obtained by Wang *et al*. [118] who, on exposing PC-12 cells to increasing concentrations of copper NPs (Cu-NPs), found a significant reduction in the levels of DA, DOPAC and HVA, probably caused by oxidative stress and enzymatic alterations. The toxicological behavior of citrate-reduced Au-NPs and Ag-NPs was studied by Love and Haynes [119] on primary cultures of adrenal medullary chromaffin cells. Treatment with 0.01–1 nM of metal-based NPs did not affect cell viability, but caused less and slower secretion of epinephrine molecules, indicating the ability of these NPs to interfere with chemical messenger delivery and the vesicular kinetic. These findings were confirmed by a subsequent study conducted on murine adrenal medullary chromaffin cells exposed to different sizes of Ag-NPs (1 nM) and to polyethilen-glycol (PEG) and heparin surface-modified Au-NPs (10 μg/mL). In fact, the results of this study showed that noble metal NPs disrupt exocytosis, typically by altering the number of molecules and release kinetics, and enhance direct disruption of the vesicle matrix [123]. The functionality of the exocytotic machinery of mouse (C57BL/6J) chromaffin cells was also severely damaged by exposure to 5–36 nM of carboxyl QDs with CdSe core and ZnS shell since these were able to impair Ca^2+^ influx and consequently compromise the overall catecholamine supply from chromaffin cells [120]. Interestingly, Ag-NP (coated with citrate or polyvinylpyrrolidone) exposure (30 μM) also impairs neurodevelopment in PC12 cells as demonstrated by a depleted emergence of the acetylcholine-phenotype and an enhanced differentiation into a DA-phenotype after treatment with citrate-coated Ag-NPs and polyvinylpyrrolidone-coated Ag-NPs, respectively [121].

In a study on fullerene C_60_, Corona-Morales *et al*. [116] observed that exposure of Wistar rat adrenal chromaffin cells to 100 μM of these NPs, remarkably increased cell survival and prevented cell death, including apoptosis. The beneficial effects observed in this study were probably due to the strong antioxidant capacity of fullerene C_60_. Conversely, the exposure of cultured mouse chromaffin cells to increasing doses (30–263 μg/mL) of MWCNTs caused a dose-dependent reduction both in cell membrane input resistance and the number of spontaneously active cells [130]. Finally, exposure of the PC12 cell line to 25–200 μg/mL of silica NPs resulted in a dose-dependent reduction in DA synthesis associated with redundant ROS generation in the cells [122].

## 6. Other Effects of Endocrine Disrupting NPs

A limited number of studies have investigated the adverse effects of Cr-NPs on the pituitary gland (see Table 5) by evaluating secretion and the levels of growth hormone (GH). The exposure of Sprague-Dawley rats to 150, 300, and 450 μg/kg of Cr-NPs did not cause significant alterations in the concentrations of pituitary-produced GH [106]. Similar results (no significant difference in GH level between the exposed and control groups) were obtained in crossbred pigs (Duroc x Landrace x Yorkshire) treated with 200 μg/kg of Cr-NPs [112], while, in a subsequent study, the same research group observed that these NPs were able to alter GH pulsatile secretion and pituitary GH-mRNA expression by inducing an increase in the mean level, lowest value, peak value and peak duration of GH secretion and by significantly improving pituitary GH-mRNA expression [124]. These conflicting results are probably due to the different experimental design of the studies. In fact, given the pulsatile nature of GH release in most mammals, a measurement technique based on a single blood sample [112] is not sufficiently sensitive to assess the effects of treatment on GH.

The secretory function of adrenal glands may also be affected by exposure to NPs as demonstrated by some *ex vivo* and *in vivo* studies carried out using NRDE-NPs (see Table 5). In fact, the treatment of male C57BL/Jcl mice with 41.73 μg/m^3^ and 152.01 μg/m^3^ of NRDE-NPs inhibited, at the highest dose, both basal and ACTH-stimulated corticosterone release by adrenal cells, while the lowest NRDE-NP concentration increased synthesis and release of the hormone following a biphasic dose response curve [96]. However, the same research group demonstrated that there were no significant differences in plasma corticosterone concentrations in adult male Fischer rats and in male C57BL/Jcl mice exposed to 15.37, 36.35 and 168.84 μg/m^3^ and to 41.73 μg/m^3^ and 152.01 μg/m^3^ of NRDE-NPs, respectively [33,96]. Conversely, an increase in serum corticosterone levels was evident in pregnant Fischer rats treated with NRDE-NPs (148.86 μg/m^3^) [90], while in immature male Fischer rats, prenatally exposed to these NPs, there was a significant reduction in serum hormone concentrations [34]. Finally, with regard to cortisol secretion, Zha *et al*. [106] showed that the administration of 150–450 μg/kg of Cr-NPs to Sprague-Dawley rats reduced plasma cortisol levels.

## 7. Impact of Endocrine Disrupting NPs on Invertebrate Species

Currently, most of the studies to assess the adverse effects of EDCs are performed on vertebrates, while significantly less attention is paid to the effects on invertebrates. However, invertebrates account for most animal biodiversity and are important in routine ecotoxicological testing and environmental monitoring [131]. Thus, understanding the adverse effects on invertebrate endocrinology caused by endocrine disrupting NPs may be useful since this species could act as sentinel organisms to evaluate the environmental impact of this category of endocrine disruptors.

Several studies that evaluated the potential endocrine disrupting activity of different types (metal- and carbon-based NPs and QDs) of NPs in *Daphnia magna*, have reported a significant impact on the reproductive health of this invertebrate. A reduced number of offspring per female was observed in *Daphnia magna* chronically treated with different sized TiO_2_-NPs at concentrations ranging from 0.01 to 100 mg/L [132]. Similar results (reduced number of offspring and complete inhibition of reproduction at the highest dose) were obtained on exposing *Daphnia magna* to 0.1–5 mg/L of Degussa P25 TiO_2_-NPs [133] or to 0.02–2 mg/L of different types of the same metal-based NPs for 21 days [134]. A significant inhibition of reproduction was also observed in *Daphnia magna* exposed to 5–500 μg/mL of Ag-NPs [135]. A study on carbon-based NPs, [136] showed that the administration of 2.5 ppm of fullerene C_60_ could delay molting and reduce offspring production in *Daphnia magna*, whereas at sub-lethal doses of exposure, Tao *et al*. [137] reported observing the accumulation of reproductive responses such as delayed maturation of the female offspring of gestating exposed daphnids and the inability to reproduce of mother daphnids after fullerene C_60_ exposure. A decrease in the total number of neonates per female was also detected for nanodiamonds at concentrations ≥ 1.3 mg/L [138]. In order to assess the estrogenic effects of QDs on this aquatic species, Kim *et al*. [139] exposed juvenile *Daphnia magna* to concentrations ranging from 0 to 30 μg/L of CdSe/ZnSe QDs coated with tri-*n*-octylphosphine oxide/gum arabic. The results of this study failed to reveal changes in VTG mRNA expression, however, the administration of 3-mercaptopropionic acid-coated CdSe/ZnSe QDs with relevant levels of UV-B, induced a dose-dependent reduction in VTG expression. In another invertebrate aquatic organism, the marine amphipod *Corophium volutator*, exposure to 0.2–1 mg/L of ZnO-NPs caused a delay in sexual maturation and reduced fecundity [140].

The endocrine disrupting effects of metal-based NPs were also observed in *Chironomus riparius* and *Drosophila Melanogaster*. Exposure of *Chironomus riparius* midge larvae to 0–1 mg/L of these NPs reduced pupation, adult emergence, the male/female ratio and egg number, probably through an up-regulation of the gonadotropin releasing hormone (GnRH) mRNA gene expression [141]. Moreover, Ag-NPs may interfere with the *Chironomus riparius* midge ecdysteroid system by altering ecdysteroid receptor mRNA expression [142]. In *Drosophila Melanogaster*, the addition to food (0.005%–0.5% *w*/*v*) of TiO_2_-NPs and Ag-NPs led to a significant decrease in developmental success and, in the case of exposure to TiO_2_-NPs, to a significant progeny loss, as shown by the decline in female fecundity. Similarly, a substantial reduction in the *Drosophila melanogaster* lifespan and fertility performance was observed after the ingestion of 0.114–467 mg/g of citrate-capped Au-NPs [143].

A number of studies have reported reproductive toxicity in worms in a terrestrial environment due to various metal oxide NPs. Five days of dietary exposure to TiO_2_-NPs (>47.9 mg/L), ZnO-NPs (1.2 and 4.6 mg/L) and Al_2_O_3_-NPs (>102 mg/L) reduced nematode *Caenorhabditis Elegans* growth, the number of eggs and offspring per worm, indicating NP-induced reproductive impairment in this species [144,145]. Comparable results that demonstrated the ability of metal-based NPs (Ag-NPs, CeO_2_-NPs, TiO_2_-NPs and ZnO-NPs) to affect the reproductive potential of *Caenorhabditis Elegans* were obtained by Roh *et al*. [146,147] and by Ma *et al*. [148]. A subsequent study carried out by Lim *et al*. [149] suggested that oxidative stress was the leading mechanism in inducing Ag-NP reproductive impairment in this invertebrate species. In addition to metal-based NPs, QDs, carbon-based and silica NPs also produced significant detrimental effects on the reproductive function of *Caenorhabditis Elegans*. In fact, egg alterations were observed after exposure to 200 nM of CdSe/ZnS or CdTe-QDs [150], while a reduction in the reproductive rate, assessed as the number of eggs per animal, was induced by treatment with 100 μg/mL of hydroxylated fullerene [151]. Furthermore, the administration of 2.5 mg/mL of silica-NPs caused a reduction in progeny and a manifestly premature cessation of progeny production due to age-related degeneration of the reproductive organs [152]. Disruption of the reproductive function related to several types of metal, metal oxide and carbon-based NPs, was also observed in *Eisenia fetida* and *Eisenia veneta* earthworms. In fact, exposure to 2.5–25 mg/kg of Au-NPs caused a dose-dependent decrease in the number of offspring [153], while acute exposure to PVP-coated Ag-NPs (801 ± 74.9 and 773.3 ± 11.2 mg/kg) [154] and treatment with 3000 and 10,000 mg/kg of Al_2_O_3_-NPs (11 nm) resulted in a lower number of cocoons [155]. Similar results were also observed in *Eisenia fetida* and *Eisenia veneta* exposed to ZnO-NPs and TiO_2_-NPs [156] and to ZnO-NPs [157], respectively. In contrast, the exposure of *Eisenia fetida* to Cu-NPs at concentrations of 5–50 mg/kg dry mass did not cause any reproductive effects [158]. With regards to carbon-based NPs, treatment of *Eisenia veneta* earthworms with double walled-CNTs led to a significant reduction in cocoon production [159] and similarly, the cocoon number diminished in *Eisenia veneta*, *Eisenia fetida* and *Lumbricus rebellus* exposed to C_60_ fullerene [159–161]. However, a study conducted by Pakarinen *et al*. [162] failed to confirm the reproductive toxicity of C_60_ fullerene in *Lumbriculus variegatus* administered with 10 and 50 mg/kg of the aforementioned carbon-based NPs.

## 8. Discussion and Conclusions

Recent advances in engineering and technology have led to the development of many types of NPs currently used in numerous industrial sectors. The rapidly developing field of nanotechnology, which is creating materials with size-dependent properties, is likely to become an important source of exposure to NPs. In fact, as more and more consumer products containing NPs become available on the market, exposure of the general population will inevitably increase. However, as with any new technology, the earliest and most extensive exposure to hazards is most likely to occur in the workplaces and workers in nanotechnology-related industries may be particularly exposed to uniquely engineered materials with innovative sizes, shapes, and physical and chemical properties. The growing NP exposure of workers and the general population to NPs has heightened concern about the potential human toxicity and environmental impact of these particles. Consequently, in the past few years, nanotoxicology has emerged as a new discipline designed to elucidate the relationship between the physical and chemical properties of NPs and the induction of toxic biological responses [163]. However, since there is still a lack of knowledge about the possible risks to human health and environmental safety posed by the expanding development and use of NPs, there seems to be an urgent need to gather information on this subject.

In this context, we have summarized the data currently available in the literature that report the adverse effects on the endocrine system caused by *in vitro* and *in vivo* exposure to different types of NPs. Our aim was to understand the risk of NP endocrine disruption, assess the underlying mechanisms that can affect endocrine function and identify research areas where further study needs to be carried out to reach a deeper understanding of the role of NPs as endocrine disruptors. Current data support the notion that different types of NPs are capable of altering the normal and physiological activity of the endocrine system (see Tables 2, 3, 4 and 5). The male and female reproductive systems are the endocrine organs that have received most attention. In the male reproductive system (see Table 2), findings showed that NPs are able to affect cell viability in gonadal tissues, testicular morphology and the process of spermatogenesis, while in the female reproductive system (see Table 3), NPs exerted cytotoxic and/or genotoxic effects on ovarian structural cells and damaged oogenesis and follicle maturation. Furthermore, NPs caused significant alterations in normal sex hormone levels in both systems. However, some studies failed to confirm these adverse effects, since no abnormalities in male and female reproductive function were found after NP exposure (see Tables 2 and 3). These conflicting results are probably due to the different intrinsic properties of NPs used in the *in vitro* and *in vivo* studies. In fact, research has shown that the physico-chemical characteristics (particle size, shape, surface area, charge, chemical properties, solubility, oxidant generation potential, and degree of agglomeration) of NPs can influence their effects on biological systems [20].

This supposition is also confirmed by the findings of some of the studies reviewed, since the physico-chemical properties of NPs seem to influence the toxic effects induced on endocrine function. For instance, NP size impacted spermatogenesis, sperm quality and testosterone levels in different ways [47,56], while surface functionalization appeared to bring about variations in both male and female reproductive NP effects [39,44,47,78,81]. Moreover, Love *et al*. [123] suggested that different NP physico-chemical properties, including size and surface coating, modulate changes in the cell communication of murine chromaffin cells exposed to different types of Ag-NPs and Au-NPs. Therefore, since the biological and toxic effects of NPs are highly dependent on their physico-chemical properties as well as on dosage, route of administration and exposure time [41], it is evident that a clear and precise evaluation of the effects of NPs as endocrine disruptors should also include a homogeneous exposure classification to ascertain exactly how the physico-chemical properties of NPs correlate with their adverse effects on the endocrine system. Useful parameters for a systematic categorization should include chemical composition, morphology, number concentration, surface area, mass concentration, weighted size distribution, state of agglomeration and surface reactivity (e.g., the ability to produce radicals and zeta potential). This is fundamental for an assessment of the endocrine toxicity of NPs, but unfortunately much of this characterization is largely lacking in current studies. For example, most available studies provide an indication of the chemical composition and size of the NPs used, while only a few of the reviewed studies go beyond this basic information to include data on other parameters such as functionalization [37,39,42–44,57,69,78,81,82,84,94,120,121,123], number concentration [33,34,49,58,60,89,96], surface area [35,36,70,79,86,88], shape [72] and degree of aggregation [85].

Furthermore, in addition to the “intrinsic” NP properties, it may be extremely useful to evaluate the properties “extrinsically” acquired by NPs when interacting with biological fluids [164]. In fact, when NPs enter a physiological environment, they can rapidly adsorb proteins forming what is known as the protein corona. The protein corona provides the NPs with a biological identity that is substantially different from their synthetic identity [165], and, by affecting interaction with biomolecules, membranes and physical barriers, leads to specific toxic effects. Consequently, it is possible that quite different nanomaterials that share similar corona properties may have similar early biological interactions [164]. Interestingly, each physiological compartment has its own distinct set of proteins that interact in a unique way with the NPs. This is an important aspect to consider when comparing *in vitro* and *in vivo* experiments, but also when evaluating *in vivo* studies in which different routes of exposure were used [166]. In fact, since the protein corona reflects the traiectory of NPs in the body, a nanomaterial that enters the blood through the lung may have a dramatically different protein corona composition and resulting physiological response than the same nanomaterial directly injected into the blood [167]. Another important issue regarding the potential role of NPs in endocrine disruption is related to the molecular mechanisms of action underlying the adverse effects observed. In several studies, oxidative stress was hypothesized as the principal damaging mechanism [37,56,71,86]. However, oxidative stress is not the only mechanism of action capable of altering the functionality of the endocrine system, since Ag-NPs have been shown to inhibit cell proliferation by disrupting the proliferation signaling cascade of spermatogonial stem cells, rather than by inducing an oxidative reaction [47]. A similar mechanism has also been reported for SiCNWs on ovarian cells [73]. Furthermore, direct DNA damage has been reported as a potential toxic mechanism in mouse and human spermatozoa in *in vitro* [45,49] and *in vivo* models [55,56]. Numerous studies have also shown that NP endocrine toxicity occurs due to its ability to modify the gene expression of proteins and enzymes involved in sex hormone biosynthesis, metabolism and release [30,33,34,59–61,86,89,90,120]. Another important mechanism of action by means of which EDCs are able to alter functions of the endocrine system is related to their ability to bind to hormone receptors. This mechanism of action may also be used by NPs and may play a significant role in the induction of endocrine effects. Indeed, the estrogenic effects exerted in mammalian cells and animal models may be dependent on NP interaction with nuclear and plasma membrane receptors, leading to activation of the estrogenic signaling pathway. For instance the findings of a study conducted by Jain *et al*. [99] suggest that the gradual and sustained release of ionic cadmium may contribute to the metalloestrogenic effects of QDs with a cadmium core. Cd^2+^ may bind to ER to trigger estrogenic effects including nongenomic activation of MAPK and PI3K as well as genomic activation, which involves ER dimerization and binding to the estrogen response element to induce gene transcription. To confirm that the estrogenic signaling induced by QDs was mediated via the estrogen receptor, the Authors found that the addition of the selective estrogen receptor antagonist, ICI 182780, completely annulled all QD-induced estrogenic effects [99].

The results of this study and the findings of Terzuoli [50], who suggested that the release of Ag^+^ under aqueous conditions could damage sperm membrane, endorse the idea that the release of ions by metallic NPs or QDs may play a relevant role in the induction of endocrine impairments in *in vitro* and *in vivo* models. Dissolution processes, consisting in the release of metallic cations, are greater in NPs than in bulk materials and are influenced by numerous factors such as reduced size, high surface-to-mass ratio, high radii of curvature, and corresponding low coordinated atoms at the surface [168]. This phenomenon is able to modify NP morphology and determine detrimental health effects [169–171]. In fact, different studies have demonstrated the correlation between NP toxicity and ions released from Ag-NPs [172–174], CdSe-QDs [175,176] and ZnO-NPs [170]. Therefore, the adverse effects on the endocrine system, potentially caused by the release of metal ions, should be more extensively evaluated in future studies. Moreover, NPs may also act by impairing the ligand-elicited signaling cascade as demonstrated by the direct interruption of insulin signaling in liver cells due to exposure to TiO_2_-NPs [110]. In this study, NPs reduced the phosphorylation on tyrosine residues of insulin receptor substrate proteins causing a decrease in the lipid messenger phosphatidylinositol 3,4,5 triphosphate wich is needed for most metabolic effects of insulin. The systematic identification of the molecular mechanisms that NPs may use to cause adverse effects on the endocrine system is particularly difficult because changes in their different physico-chemical properties may trigger different mechanisms of toxic action. Consequently, once more, accurate physico-chemical characterization of NPs seems essential in order to obtain a better understanding of the mechanisms of action.

Toxicity testing of NPs using *in vitro* or *in vivo* assays is aimed at identifying a potential risk by establishing dose-response relationships that characterize this type of hazard. However, toxicological studies (even those that have investigated effects on the endocrine system), frequently use excessive and unrealistically high mass doses of NPs [177]. It is therefore debatable whether the findings observed in these studies can be extrapolated to refer to humans in realistic, lower exposure scenarios since the applied doses have little relevance to real-world conditions. Although it is not easy to determine a realistic exposure dose to be used in these toxicological studies, as available data on exposure levels in workers and the general population are still very limited [20], it seems clear that future studies need to focus on the potentially adverse effects on the endocrine system of low-level NP exposure especially through the use of exposure doses similar to those identified in environmental monitoring studies. Furthermore, considering that in recent years additional metrics such as number concentration and surface area are being taken into account in the assessment of exposure to NPs [178], it would be interesting to express exposure doses also in terms of number concentration and surface area as has already been done in some studies [33–36,49,58,60,70,79,86,88,89,96].

The results of some studies involving the nature of the dose-response curve indicate that exposure to NPs may exert a hormetic response in endocrine organs. In fact, when evaluating changes in testosterone levels caused by NRDE-NPs, Li *et al*. [33] showed a clear inverted-U dose-response curve, while Ag-NPs were able to affect the TH-action on TRβ and RLKI transcripts according to an inverted-U hormesis response [105]. Hormesis has been observed in a number of studies that investigated the health effects of NPs and recently, Iavicoli *et al.* [24] have reviewed studies that reveal similar hormetic dose-responses in a variety of nanomaterials (e.g., single walled carbon nanotubes; silver molybdenum and aluminum nanoparticles, graphite nanofibers, titanium dioxide nanoparticles). However, at present, little is known of the reasons underlying possible NP induction of hormesis for some endpoints. Further research is therefore needed to better understand the physico-chemical properties of NPs that may induce hormesis, and the hazard endpoints that are most likely to be involved. It should be noted that factors such as aggregation and agglomeration can significantly influence NP characteristics in exposure and hazard studies [26]. In this context, a possible explanation of NP-induced hormesis may also lie in the aggregation of NPs at higher concentrations. Correct evaluation of this phenomenon therefore requires appropriate characterisation of the NPs in the test medium, since these can greatly influence the extent of particle agglomeration. Finally, to verify the actual presence of hormesis in NP dose-responses and interpret study findings, it might be helpful to apply the methodology suggested by Nascarella and Calabrese [24,179]. Summing up, future studies could provide invaluable information by ascertaining whether this particular phenomenon also occurs in other endocrine organs, or whether it is a result of exposure to other, different types of NPs.

A critical issue concerning exposure to EDCs is the possibility that these substances may cause not only somatic but also intergenerational adverse effects on the endocrine system. In fact, in recent years, numerous studies have provided evidence that fetal or perinatal exposure to EDCs results in disturbed endocrine functions, especially in the sexual development of offspring [9]. In this context, some study findings have revealed that *in utero* exposure to CB-NPs and NRDE-NPs can also lead to testicular morphological and functional alterations in the male offspring of rats and mice [34,38]. Whether the toxic effects on pup gonads derived from direct NP action or were the result of alterations induced in the maternal endocrine/reproductive system is an open question. Therefore, since exposure to EDCs during critical periods of development may cause adverse consequences later in life, and since NPs may play a role as EDCs, future studies on the potential endocrine toxicity of NPs should also take into account the age, period of exposure and mechanisms of action of possible epigenetically-mediated intergenerational effects.

Of all the studies on different types of NPs, it is those that have investigated possible adverse effects on the endocrine system due to DE-NPs that have provided the most intriguing results from an environmental point of view. DE-NPs are the most common incidental combustion-derived NPs in the urban environment and can also occur in occupational settings [33]. The results of the studies reviewed [33,39,59–61,90,96] point to an endocrine disrupting activity on the part of DE-NPs, although lack of evidence regarding the relationship between the exposure doses used in the aforementioned studies and real environmental concentrations make it difficult to extrapolate these results to humans. Furthermore, since environmental contamination is generally due to a mixture of compounds, the disrupting effects of DE-NPs may be due to chemical substances around or attached to them which then exert an additive, synergic or antagonistic influence. It follows that further investigation on the toxicological profile of DE-NPs and the environmental pollutant mixture that generally envelops them is urgently needed in order to clarify their impact on endocrine function.

The results of studies conducted on invertebrates provide valuable information for assessing the biota impact of NPs as endocrine disruptors. In fact, several parameters such as invertebrate growth and molting, changes in vitellogenin levels, and reduced fecundity are useful indicators of endocrine disruption activity. However, further research is also needed in this area to identify the endocrine disrupting mechanisms and thereby determine the specific biomarkers that would be effective in measuring these alterations.

Finally, a complete and systematic assessment of the endocrine disrupting activity of NPs should be based on an analysis of the adverse effects they exert on the entire endocrine system. Currently, there is a lack of balance between studies that have evaluated their toxic effects on the male and female reproductive system and studies that have investigated the same effects on other endocrine organs such as the thyroid, neuroendocrine system, pituitary and adrenal glands (see Table 5).

In conclusion, the data currently available indicate that several types of NPs can adversely affect the endocrine system, and in particular the male and female reproductive system. In fact, the results of the studies presented in this review suggest that NPs are able to disrupt the endocrine system by exerting cytotoxic effects and damaging the costituent cells of endocrine organs, thus influencing endocrine homeostasis, altering hormone byosinthesis, metabolism and release and interfering with the ligand elicited signaling. However, a more critical assessment of these findings suggests the need to interpret these results with caution since the information on the potential interations and toxicity of NPs on the endocrine system is quite limited and in some cases conflicting. This means it is not possible to to reach a broad and shared consensus on the role of NPs as endocrine disruptors on the basis of data currently available and further investigation is clearly required. Moreover, the current difficulty in reaching definitive conclusions on this topic is due to the heterogeneity of experimental study designs and to the numerous variables that can influence the toxicological profile of NPs. Obtaining an overview of results is complicated and challenging when quite different cell lines or laboratory animals, routes, times and doses of exposure (most of which are excessively high or not representative of a real human exposure) have been used in the studies reviewed. Clearly there is a need to establish a systematic and rational approach in the design of future studies. Moreover, the adverse effects of NPs on human health often depend on the chemical structure, shape, size, agglomeration state, surface area and functionalization of NPs. These are therefore among the factors to be considered in studying nanotoxic effects on the endocrine system. Finally, a key issue such as the molecular mechanisms underlying the potential endocrine toxicity of NPs is still largely unknown or unexplored.

It is hoped that this systematic review will provide helpful information and a stimulus for discussion on aspects that should be the focus of future studies. In our opinion, it is necessary to (i) carry out *in vitro* and *in vivo* studies using NPs whose chemical and physical properties have been characterized in a clear and detailed manner, (ii) clarify all the different potential molecular mechanisms of action underlying the adverse effects of NPs on endocrine organs, including those responsible for intergenerational effects (iii) study the biological responses of the endocrine system in relation to low-level exposures to NPs (iv) identify the presence of hormesis and (v) evaluate the endocrine disrupting effects of NPs on the entire endocrine system.

## Figures and Tables

**Table 1:** Categories, criteria of classifications and numbers of chemical substances included in the Endocrine Disrupter Priority list (EDPL). The constitution and the integration of the EDPL is carried out by a process of analysis which consists of three steps. First of all, a working list of suspected endocrine disruptors chemicals is compiled, based on the information provided by various organizations and on the analysis of the literature (step 1). Subsequently the available information is reviewed to identify those chemicals that are more relevant, in term of exposure, for both humans and animals (step 2). Finally, the identified chemicals are studied to determine the strength of evidence for endocrine disruption and to enroll them in a specific category (step 3). Addition and removal of chemicals may be required in response to either developments in scientific knowledge or changes in chemical usage patterns.

**Type of category**	**Category 1**	**Category 2**	**Category 3a**	**Category 3b**	**References**
**Criteria of classification**	At least one *in vivo* study providing clear evidence of endocrine disruption in an intact organism	Potential for endocrine disruption. *In vitro* data indicating potential for endocrine disruption in intact organisms. Also includes effects *in vivo* that may, or may not be endocrine disruption-mediated.	No scientific basis for inclusion in list. Endocrine disruption studies available but no indications of endocrine disruption effects.	Substances with no or insufficient data gathered	
**Number of chemical substances in each category**	194	125	23	86	[15–18]

**Table 2:** Effects of NPs on the male reproductive system.

Type of study	Type and physico-chemical properties of NPs	Experimental protocol	Cell line/laboratory animals	Results	References
**Alterations and impairment of testicular structure**					
*In vitro*	TiO_2_-NPs (25–70 nm); CB-NPs (14 nm); DE-NPs	Treatment with 0–1000 μg/mL for 24 and 48 h	Mouse testis Leydig cell line TM3	Internalization of NPs; Dose-dependent reduction of cell viability after 48-hour TiO_2_-NP exposure.	[30]
*In vitro*	75% rutile and 25% anatase TiO_2_-NPs (24.5 nm)	Treatment with 0.5–50 μg/mL for 4, 24 and 48 h	RTG-2 cells	Significant cytotoxicity only at the highest concentration.	[31]
*In vitro*	CeO_2_-NPs (10 nm and 20–25 nm)	Treatment with 0–100 μg/mL for 24 and 72 h	RTG-2 cells, derived from rainbow trout (*Oncorhynchus mykiss*) gonadal tissue	No cytotoxic effects.	[32]
*In vivo*	NRDE-NPs—Average of mode diameter and number concentrations for low, middle and high doses were 22.48, 26.13 and 27.06 nm; 2.27 × 10^5^, 5.11 × 10^5^ and 1.36 × 10^6^, respectively	Inhalation exposure to low (15.37), middle (36.35) and high (168.84) μg/m^3^ doses for 4, 8, or 12 weeks (5 h/day, 5 days/week)	Fischer F344 rats	Seminiferous tubules showed degenerative and necrotic changes, interstitial edema, desquamation of the seminiferous epithelium, and loss of spermatozoa.	[33]
*In vivo*	NRDE-NPs—Average of mode diameter and number concentrations were 26.81 nm and 1.83 × 10^6^, respectively	Inhalation exposure to 148.86 μg/m^3^ for 5 h daily for 19 gestational days	Pregnant Fischer F344 rats	Loss of germ cells in the seminiferous tubules of male offspring.	[34]
*In vivo*	TiO_2_-NPs (anatase form, particle size of 25–70 nm, surface area 20–25 m^2^/g)	Subcutaneous injections of 100 μL of 1mg/mL solution on 4 gestational days	Pregnant Slc:ICR mice	Disruption of seminiferous tubules; Decreased numbers of Sertoli cells.	[35]
*In vivo*	CB-NPs (14, 56 and 95 nm with a surface area of 300, 45 and 20 m^2^/g, respectively)	Intratracheal administration of 0.1 mg/kg body weight for 10 times every week	ICR mice	Partial vacuolization of the seminiferous tubules; The adverse effects depend on particle mass rather than particle number.	[36]
*In vivo*	Carboxylate-functionalized (diameter of 20–30 nm, length 0.5–2.0 μm) and Amine-functionalized (diameter of 20–30 nm, length 0.5–2.0 μm) MWCNTs	Mice were randomly divided into 12 groups (15-day single dose, 15-day multi-dose, 60-day multi-dose, and 90-day multi-dose groups). Mice were given injections (5 mg/kg) via the tail vein once (single dose) or every 3 days for 5 times	BALB/c mice	Reduction in the thickness of the germinative layer; Reduced spermatogonia number; Partial disappearance or vacuolization of Sertoli cells; Induction of oxidative stress.	[37]
*In vivo*	CB-NPs (14 nm)	Intratracheal administration of 0.2 mg/kg body weight on days 7 and 14 of gestation	Pregnant ICR mice	Partial vacuolization of seminiferous tubules; Reduction of cellular adhesion of seminiferous epithelia.	[38]
*In vivo*	mPEG@Au-NP and PEG-NH_2_@Au-NP (14 nm)	Intravenous injection of 45 and 225 mg/kg of mPEG@Au-NP and 45 mg/kg of PEG-NH_2_@ Au-NP at 48 h intervals for 5 days	ICR mice	Penetration and accumulation of NPs in the testes; No testicular morphological changes; No germ cell apoptosis.	[39]
*In vivo*	Amorphous silica particles (nSP70, 70-nm diameter; nSP300, 300-nm diameter)	Intravenous injection of 0.4 and 0.8 mg of nSP70	BALB/c mice	nSP70 can penetrate the bloodtestis barrier and the nuclear membranes of spermatocytes without producing apparent testicular injury.	[40]
**Alterations and impairment of spermatogenesis**					
*In vitro*	Fullerenol	Treatment with fullerenol (1, 10 and 100 μmol) for 3 h.	Epididymal sperm samples collected from the fresh epididymis of adult goats	Protective effect, by increasing the activities of antioxidant enzymes and decreasing lipid peroxidation, on the potential oxidative stress damage on spermatozoa.	[41]
*In vitro*	Silver (15 nm), molybdenum (30 nm), and aluminum (30 nm)	Treatment with 5, 10, 25, 50, and 100 μg/mL culture medium for 48 h	C18-4 spermatogonial stem cell line	concentration-dependent toxicity for all types of particles tested; Silver nanoparticles were the most toxic while molybdenum trioxide (MoO_3_) nanoparticles were the least toxic.	[42]
*In vitro*	Magnetic iron oxide NPs (Fe3O_4_-NPs) coated with poly(vinyl alcohol)	Treatment with 7.35 mM for 80 min and 4 h	Bovine sperm cells	No effects on the motility and the ability to undergo the acrosome reaction.	[43]
*In vitro*	Eu_2_O_3_-NPs (30 ± 10 nm); EuOH_3_-NPs conjugated with polyvinyl alcohol or polyvinyl piyrolidone (15.4 ± 3 nm and 9.3 ± 3 nm, respectively)	1 mL of washed sperm cells was incubated for 24 h at 39 °C with 2.5 mg/mL of Eu_2_O_3_-NPs or of EuOH_3_-NPs	Bovine sperm cells	Loss of bovine spermatozoa motility after exposure to Eu_2_O_3_-NPs; No effects on motility and the ability to undergo acrosome reaction after exposure to EuOH_3_-NPs.	[44]
*In vitro*	TiO_2_-NPs and ZnO-NPs (40–70 nm)	Treatment with 3.73–59.7 μg/mL of TiO_2_-NPs and 11.5–93.2 μg/mL of ZnO-NPs	Human spermatozoa	Concentration - dependent induction of sperm DNA damage.	[45]
*In vitro*	Au-NPs (9 nm)	Analyses were conducted on a mixture of 500 μL of Au-NPs (44 ppm) solution and semen	Human spermatozoa	25% of sperm were not motile; Penetration of Au-NPs into the sperm head and tails; Fragmentation of sperm.	[46]
*In vitro*	Hydrocarbon-coated silver (Ag-HC) nanoparticles of 15, 25, and 80 nm diameters and Polysaccharide-coated silver (Ag-PS) nanoparticles of 10, 25–30, and 80 nm diameters	Treatment with 5, 10, 25, 50, and 100 μg/mL culture medium for 24 h	C18-4 spermatogonial stem cell line	At concentrations ≥ 10 mg/mL, Ag-NPs induced a significant decline in SSC proliferation, which was also dependent on their size and coating; At a concentration of 10 mg/mL, Ag-NPs specifically interact with Fyn kinase downstream of Ret and impair SSC proliferation.	[47]
*In vitro*	Au-NPs (5–65 nm)	Treatment with 0.5–50 μM for 2 h	Bovine spermatozoa	22% loss in sperm motility after exposure to the highest dose.	[48]
*In vitro*	Au-NPs (~2.5 nm in diameter)	Treatment with concentrations of 0.5 × 10^15^ or 1 × 10^15^ particles/mL for 20 and 40 min	Epididymal sperm samples collected from the epididymis of hybrid mice CBA × C57B1/6	Au-NPs possess spermatotoxicity, causing an inhibition of chromatin decondensation process in gametes.	[49]
*In vitro*	Ag-NPs (65 nm)	Aliquots of total semen were incubated at 37 °C for 60 min and 120 min at the concentration of 125, 250 and 500 μM	Semen samples obtained from 10 healthy donors	Decline in sperm motility and viability even at the lowest concentration used; The cytotoxic effect occurs in a dose dependent manner; Sperm viability was always higher than the percentage of sperm motility meaning that spermatozoa were viable but immotile.	[50]
*In vitro*	Ag-NPs (20 nm) and TiO_2_-NPs (21 nm)	Cells were exposed for 24, 48 and 72 h to 10, 50 and 100 μg/mL, equivalent to 7.8, 15.6 and 31 μg/cm^2^, respectively	Ntera2 (NT2, human testicular embryonic carcinoma cell line) and primary testicular cells from C57BL6 mice of wild type (WT) and 8- oxoguanine DNA glycosylase knock-out (KO, mOgg1−/−) genotype	Ag-NPs are more cytotoxic and cytostatic compared to TiO_2_-NPs, causing apoptosis, necrosis and decreased proliferation in a concentration- and time-dependent manner.	[51]
*In vivo*	TiO_2_-NPs	Intraperitoneal injection of 200 and 500 μg/kg every other day for five times	ICR mice	No significant changes in the low dose group; Reduced sperm density and motility, increased sperm abnormality and germ cell apoptosis in the high dose group.	[52]
*In vivo*	TiO_2_-NPs (anatase form, particle size of 25–70 nm, surface area 20–25 m^2^/g)	Subcutaneous injections of 100 μL of 1 mg/mL solution	Pregnant Slc: ICR mice	Decrease of DSP and epidydimal sperm motility in male offspring.	[35]
*In vivo*	CB-NPs (14, 56 and 95 nm with a surface area of 300, 45 and 20 m^2^/g, respectively)	Intratracheal administration of 0.1 mg/kg body weight every week for 10 times	ICR mice	The DSP decreased by 33% in the 14 nm CB group, by 33% in the 56 nm CB group and by 23% in the 95 nm CB group.	[36]
*In vivo*	Carboxylate-functionalized (diameter of 20–30 nm, length 0.5–2.0 μm) and Amine-functionalized (diameter of 20–30 nm, length 0.5–2.0 μm) MWCNTs	Mice were randomly divided into 12 groups (15-day single dose, 15-day multi-dose, 60-day multi-dose, and 90-day multi-dose groups). Mice were given injections (5 mg/kg) via the tail vein once (single dose) or every 3 days for 5 times	BALB/c mice	No alterations in the total sperm concentration, motility, percentage of abnormal semen and loss of acrosome integrity were detected in the treated mice.	[37]
*In vivo*	CB-NPs (14 nm)	Intratracheal administration of 0.2 mg/kg body weight of CB-NPs on days 7 and 14 of gestation	Pregnant female ICR mice	The DSP in the fetal carbon nanoparticle-exposed mice decreased by 47% at the age of 5 weeks, by 34% at the age of 10 weeks, and by 32% at the age of 15 weeks.	[38]
*In vivo*	C_60_ fullerene (3–36 nm)	Rats received distilled water containing aqueous solutions of C_60_HyFn at concentration of 4~μg/kg daily for 5 weeks	Healthy and streptozotocin-induced diabetic male Wistar albino rats	No significant toxic effects in testicular tissues were observed; In the diabetic rats, C_60_ fullerene increased sperm motility, epididymal sperm concentration and decreased the abnormal sperm rate, showing an important anti-oxidant activity.	[53]
*In vivo*	Dimercaptosuccinic acid coated Fe_3_O_4_-NPs (3–9 nm)	Intraperitoneal injection in a single dose of 50, 100, 200 and 300 mg/kg	Female pregnant Balb/C mice	In male infants of mothers treated with doses higher than 50 mg/kg a significant decrease in spermatogonia, spermatocytes, spermatids and mature sperm was observed.	[54]
*In vivo*	TiO_2_-NPs (33.2 ± 16.7 nm)	Administration by oral gavage of 40, 200 and 1000 mg/kg with volume of suspension 10 mL/kg of mouse weight	Male CBAxB6 mice	Increase of the testis apoptotic index and of the frequency of round spermatidis with two or more nuclei.	[55]
*In vivo*	Ag-NPs with a nominal diameter of 20 ± 5 nm	Intravenous injection with a single dose (5 mg/kg or 10 mg/kg) of Ag-NPs	Wistar rats	Decreased epididymal sperm count and increased DNA damage in germ cells at 24 h post-exposure; The most severe adverse effects were caused by the smaller particles.	[56]
*In vivo*	mPEG@Au-NP and PEG-NH_2_@Au-NP (14 nm)	Intravenous injection of 45 and 225 mg/kg of mPEG@Au-NP and 45 mg/kg of PEG-NH_2_@Au-NP at 48 h intervals for 5 days	ICR mice	No abnormalities in terms of irregular shapes or forms were observed in the sperm collected from the cauda epididymis of animals.	[39]
*In vivo*	Nude short MWCNTs (50–200 nm) and synthesized functionalized MWCNTs with polyethylene glycol (PEG) (s-MWCNTs-PEG).	Intravenous administration with a single dose of 100 μg/kg body weight	Kunming mice	No toxicity of these nanotubes on male mouse sperm production or mutagenesis in the long term exposure was observed.	[57]
*In vivo*	Powder of nanoparticulate TiO_2_ (primary particle size was 20.6 nm, in the exposure atmosphere the particle number concentration was 1.7 × 10^6^ particles/cm^3^ and the major particle size mode was 97 nm); nanosized CB (particle size was 14 nm)	Mice were exposed by whole body inhalation, 1 h/day from gestation day 8 to 18, to 42 mg/m^3^ of aerosolized powder of nanoparticulate TiO_2_; mice were intratracheally instilled four times during gestation, (days 7, 10, 15 and 18) with 67 μg/animal of nanosized CB	Pregnant female C57BL/6J mice	TiO_2_-NPs tended to reduce sperm count in the F1 generation; There was no effect on sperm production in the F2 generation originating after TiO_2_-NPs exposure; F2 offspring, whose fathers were prenatally exposed to CB-NPs, showed lowered sperm production.	[58]
**Disruption of normal levels of sex hormones**					
*In vitro*	TiO_2_-NPs (25–70 nm); CB-NPs (14 nm); DE-NPs	Treatment with 0–1000 μg/mL for 24 and 48 h	Mouse testis Leydig cell line TM3	Induction of StAR gene expression.	[30]
*In vitro*	NRDE-NPs	Mice were exposed to 152.01 μg/m^3^ of NRDE-NPs for 8 weeks	Interstitial testicular cells, dissected from male C57BL/Jcl mice	Testosterone production in interstitial testicular cells was significantly increased both with and without human chorionic gonadotropin stimulus.	[59]
*In vivo*	NRDE-NPs—Averages of mode diameter and number concentrations for low, middle and high doses were 22.48, 26.13 and 27.06 nm; 2.27 × 10^5^, 5.11 × 10^5^ and 1.36 × 10^6^, respectively	Inhalation exposure to low (15.37), middle (36.35) and high (168.84) μg/m^3^ doses for 4, 8, or 12 weeks (5 h/day, 5 days/week)	Fischer F344 rats	Exposure to low and medium concentrations of NRDE-NPs significantly increased plasma and testicular testosterone, whereas exposure to a high concentration did not (inverted-U dose-response); No alterations in LH and FSH production were observed.	[33]
*In vivo*	NRDE-NPs—Averages of mode diameter and number concentrations were 26.81 nm and 1.83 × 10^6^, respectively	Inhalation exposure to 148.86 μg/m^3^ for 5 h daily for 19 gestational days	Pregnant Fischer F344 rats and male offspring	Decreased FSH and testosterone and increased ir-inhibin levels were observed in the offspring.	[34]
*In vivo*	NRDE-NPs—Averages of mode diameter and number concentrations for low, middle and high doses were 22.48, 26.13 and 27.06 nm; 2.27 × 10^5^, 5.11 × 10^5^ and 1.36 × 10^6^, respectively	Inhalation exposure to low (15.37), middle (36.35) and high (168.84) μg/m^3^ doses for 4, 8, or 12 weeks (5 h/day, 5 days/week)	Fischer F344 rats	Low and medium exposure to NRDE-NPs significantly increased StAR- and P450scc-mRNA and their protein expressions in the testis of rats, in which the elevation pattern was very similar to that of plasma testosterone levels.	[60]
*In vivo*	CB-NPs (14, 56 and 95 nm with a surface area of 300, 45 and 20 m^2^/g, respectively)	Intratracheal administration of 0.1 mg/kg body weight for 10 times every week	ICR mice	Increased testosterone levels.	[36]
*In vivo*	Carboxylate-functionalized (diameter of 20–30 nm, length 0.5–2.0 μm) and Amine-functionalized (diameter of 20–30 nm, length 0.5–2.0 μm) MWCNTs	Mice were randomly divided into 12 groups (15-day single dose, 15-day multi-dose, 60-day multi-dose, and 90-day multi-dose groups). Mice were given injections (5 mg/kg) via the tail vein once (single dose) or every 3 days for 5 times	BALB/c mice	MWCNT exposure did not alter plasma levels of testosterone, LH, and FSH.	[37]
*In vivo*	CB-NPs (14 nm)	Intratracheal administration of 200 μg of CB-NPs on days 7 and 14 of gestation	Pregnant female ICR mice	No significant increase in testosterone levels was observed in fetal carbon nanoparticle- exposed mice.	[38]
*In vivo*	NRDE-NPs	Mice were exposed to 152.01 μg/m^3^ of NRDE-NPs for 8 weeks	C57BL/Jcl mice	Increased levels of testosterone; This result was related to the enhanced expression of genes involved in testicular cholesterol synthesis, such as HMG-CoA, LDL-R, SR-B1, PBR, P450scc, P450 17α, and 17β-HSD.	[59]
*In vivo*	mPEG@Au-NP and PEG-NH_2_@Au-NP (14 nm)	Intravenous injection of 45 and 225 mg/kg of mPEG@Au-NP and 45 mg/kg of PEG-NH_2_@ Au-NP at 48 h intervals for 5 days	ICR mice	The administration of PEG-NH2@Au-NP caused a significant increase in testosterone levels; No alterations in LH and FSH plasma values were detected.	[39]
*In vivo*	NRDE-NPs	Inhalation exposure to low (38 ± 3 μg/m^3^) and high (149 ± 8 μg/m^3^) NRDE-NP concentrations	Fischer F344 rats	High and low NRDE-NP concentrations increased plasma and testicular testosterone levels.	[61]

Ag-HC-NPs, Hydrocarbon-coated silver Nanoparticles; Ag-NPs, Silver Nanoparticles; Ag-PS-NPs Polysaccharide-coated silver Nanoparticles; Au-NPs, Gold Nanoparticles; C_60_HyFn, hydrated C_60_ fullerene; CB, Carbon Black; CB-NPs, Carbon Black Nanoparticles; CeO_2_-NPs, Cerium Oxide Nanoparticles; DE-NPs, Diesel Exhaust Nanoparticles; DSP, Daily Sperm Production; Eu_2_O_3_-NPs, Europium Oxide Nanoparticles; EuOH_3_-NPs, Europium Hydroxied Nanoparticles; Fe_3_O_4_-NPs, Iron Oxide Nanoparticles; FSH, Follicle Stimulating Hormone; HMG-CoA, 3-hydroxy-3-methylglutaryl-coenzyme A; LDL-R, Low Density Lipoprotein Receptor; LH, Luteinizing Hormone; mPEG@Au-NP, ω-methoxy poly(ethylene glycol) capped gold-NPs; MoO_3_, molybdenum trioxide; MWCNTs, Multi Walled Carbon Nanotubes; NRDE-NPs, Nanoparticle-Rich Diesel Exhaust Nanoparticles; nSP, amorphous nanosilica particles; P450 17α, Cytochrome P450 17α; P450 17β-HSD, Cytochrome P450 17β-HydroxySteroid Dehydrogenase; P450scc, Cytochrome P450 side-chain cleavage; PBR, Peripheral-type Benzodiazepine Receptor; PEG-NH2@Au-NP, ω-aminoethyl poly(ethylene glycol) capped gold-NPs; s-MWCNTs-PEG, synthesized functionalized MWCNTs with polyethylene glycol; SR-B1, Scavenger Receptor class B type 1; SSC, Spermatogonial Stem Cells; StAR, Steroidogenic Acute Regulatory protein; TiO_2_, Titanium Dioxide; TiO_2_-NPs, Titanium Dioxide nanoparticles; ZnO-NPs, Zinc Oxide Nanoparticles.

**Table 3:** Effects of NPs on the female reproductive system.

Type of study	Type and physico-chemical properties of NPs	Experimental protocol	Cell line/laboratory animals	Results	References
**Effects on ovarian cells**					
*In vitro*	Anatase TiO_2_-NPs (30 nm)	Treatment with increasing concentrations of TiO_2_-NPs (0–100 μg/mL) for 24 h	CHO-K1	Reduction of cell viability according to the increasing dose applied and the increasing ROS concentration induced.	[68]
*In vitro*	TiO_2_-NPs (anatase-80% rutile-20% with organic coating, ~21 nm; anatase-80% rutile-20% doped with di-iron trioxide, ~21 nm; anatase-80% rutile-20%, ~21 nm; rutile 100% with inorganic and organic coating, 14 nm; anatase 100% with inorganic coating, 60 nm; rutile 100% with inorganic and organic coating, 20 nm; rutile 100% with inorganic and organic coating, 15 nm; rutile 100% with inorganic coating, 20–22 nm)	Exposure for 3 h to different concentrations of TiO_2_-NPs (800, 1950, 3000, 5000 μg/mL)	CHO-WBL	None of the titanium dioxide particles tested induced any increase in chromosomal aberration frequencies either in the absence or presence of UV.	[69]
*In vitro*	TiO_2_-NPs (rutile-79% anatase-21%, particle size: 140 ± 44 nm, surface area: 38.5 m^2^/g)	Cytogenetic evaluations were conducted exposing cells to 750, 1250, and 2500 μg/mL for the 4 h non-activated test condition, to 62.5, 125, and 250 μg/mL, for the 4 h activated test condition, and to 25, 50, and 100 μg/mL for the 20 h non-activated test condition.	CHO	Genotoxicity tests demonstrated that TiO_2_-NPs were negative in *in vitro* mammalian chromosome aberration test with Chinese hamster ovary cells; TiO_2_-NPs did not induce structural or numerical chromosome aberrations in this study.	[70]
*In vitro*	Anatase TiO_2_-NPs (10–20, 50–60 nm) and rutile TiO_2_-NPs (50–60 nm)	Treatment with increasing concentrations of TiO_2_-NPs (25–325 μg/mL) for 24 h	CHO	Results showed toxic effects on CHO cells with a toxic order as 10–20 nm anatase > 50–60 nm anatase > 50–60 nm rutile, which was consistent with that of 8-OHdG generation and DNA strand breaking activities.	[71]
*In vitro*	TiO_2_-NPs (shape: complex; average particle sizes: 20 ± 7 nm; specific surface area: 142 m^2^/g) and Al_2_O_3_-NPs (shape: spherical; average particle sizes: 28 ± 19 nm; specific surface area: 39 m^2^/g)	Treatment with increasing concentrations of NPs (0.5, 1, 5, 10, 25, 50 and 100 μg/mL) for 24 h	CHO-K1	Results showed a dose-related cytotoxic effect manifested after 24 h by changes in lysosomal and mitochondrial dehydrogenase activity; The cytotoxic effects induced by Al_2_O_3_-NPs were weaker than those exerted by TiO_2_-NPs, and began at higher concentrations; Genotoxic effects were shown by MN frequencies which significantly increased at 0.5 and 1 μg/mL TiO_2_ and 0.5–10 μg/mL Al_2_O_3_; SCE frequencies were higher for cells treated with 1–5 μg/mL TiO_2_-NPs.	[72]
*In vitro*	SiCNW (diameter of 80 nm; chemical composition: Si to C ratio close to 1:1)	Exposure to different concentrations (0.5, 1.0, 5.0 and 10.0 μg/mL) of SiCNWs for 1, 3, and 5 days	CHO	CHO cells in contact with SiCNWs had significantly lower reproduction rates and genomic instability; Activation of the MAPKs cellular signaling pathway and over-expression of COX-2 were the main toxicity mechanisms; MN ratio rose sharply with increasing SiCNWs concentrations and very significant differences were found after exposure to 5 and 10 μg/mL SiCNWs.	[73]
*In vitro*	Calcium phosphate-NPs (20–30 nm, less than 10% particles were greater than 100 nm)	Cells were divided into different groups of exposure: control (treated with 4-androstene-3,17-dione), group II (treated with 10 μM of Calcium phosphate-NPs and 4-androstene-3, 17-dione) and group III (treated with 100 μM of Calcium phosphate-NPs and 4-androstene-3, 17-dione)	Granulosa cells collected from infertilite women	Increased percentage of cells in S phase; Increased apoptotic rate after the cells were treated with 100 μM of calcium phosphate-NPs for 48 h.	[74]
*In vitro*	MWCNTs (diameter ~10 nm)	In order to observe if the MWNT sheets have any toxic effect on cells, CHO cells were allowed to grow on the substrates until about 90% confluence	CHO	No toxicity was observed; The cells grew well and aligned and only a few cells elongated along the axis of the MWCNT bundles.	[75]
*In vitro*	Anatase TiO_2_-NPs (<25 nm)	Cells were maintained under exponential growth conditions and were continuously exposed for 1, 2, or 60 days to either 0, 10, 20, 40, 100 and 200 μg/mL of NPs	CHO-K1	Cell viability or proliferative ability was negatively affected by acute exposure at concentrations of 100 μg/mL and 200 μg/mL as measured by cell counts and colony formation; No genotoxic effects.	[76]
*In vitro*	Anatase TiO_2_-NPs (<25 nm)	Treatment with different concentrations of NPs (0, 25, 50, 100 and 200 μg/mL). Cells were incubated for 24 h and 48 h.	CHO-K1	Based on the trypan blue assay, viability decreased at the two higher concentrations; The decrease in cell viability using the MTT assay was only observed at the highest concentration and at 48 h incubation.	[77]
*In vitro*	Naked mesoporous silica NPs (10 nm and 50 nm); carboxyl or amine modified mesoporous silica NPs; carboxyl-modified polystyrene-NPs (30 nm); polystyrene-NPs functionalized with amine groups (50 nm)	Exposure of NIH-OVCAR3 for 48 h to 30 and 75 μg/mL of naked mesoporous silica NPs (10 nm); exposure of NIH-OVCAR3 and SKOV3 cells for 1 and 24 h to 20 μg/mL of naked mesoporous silica NPs (50 nm) and of carboxyl or amine modified mesoporous silica NPs; exposure of NIH-OVCAR3 and SKOV3 cells for 24 and 48 h to 75 μg/mL of carboxyl-modified polystyrene-NPs and polystyrene-NPs functionalized with amine groups	Ovarian NIHOVCAR3 epithelial cancer cells; SKOV3 cancer cells	No altered cell morphology, metabolism or cell loss was detected in NIH-OVCAR3 and SKOV3 cells treated with naked mesoporous silica NPs or carboxyl or amine modified mesoporous silica NPs; Evident internalization of the NPs could be observed. Carboxyl-modified polystyrene-NPs were not toxic for NIH-OVCAR3 and SKOV3 cells; Polystyrene NPs functionalized with amine groups showed significant cytotoxic effects.	[78]
*In vitro*	MWCNTs (average diameter: 67 nm; surface area: 26 m^2^/g; carbon purity: 99.79 wt%)	Exposure for 24 h to 1, 10 and 100 μg/mL of NPs	MARCO-transfected CHO-K1 cells	MWCNTs are highly cytotoxic; The toxicity of MWCNTs may be due to the incomplete inclusion of these NPs by the membrane structure.	[79]
**Effects on oogenesis and follicle maturation**					
*In vitro*	TiO_2_-NPs (25 nm)	Exposure to increasing concentrations (12.5–50 μg/mL) of TiO_2_-NPs	Rat preantral follicles	TiO_2_-NPs inhibited rat follicle development and oocyte maturation; Survival rate of follicles, formation rate of antral follicles and release rate of cumulus-ocyte cell complexes were dose-dependently decreased.	[80]
*In vitro*	CdSe-core-QDs and ZnS-coated CdSe QDs (3.5 nm)	Exposure for 24 h to 0, 125, 250 and 500 nM of CdSe-core-QDs and to 500 nM of ZnS-coated CdSe QDs	Cumulus-oocyte complexes collected from female ICR mice	CdSe-core-QDs, but not ZnS-coated CdSe QDs, induced dose-dependent decrease in oocyte maturation rate, reduced fertilization, impairment of cell proliferation and dose-dependent enhanced blastocyst apoptotic rate.	[81]
*In vitro*	Lysine coated CdSe/CdS/ZnS QDs (~20 nm)	Exposure for 4, 8, 16 and 24 h to 5.78 nmol/L and 29.8 nmol/L of Lysine coated CdSe/CdS/ZnS QDs	Immature oocytes of 28 days Kunming mice	Oocyte maturation process has high susceptibility for disturbances induced by CdSe/CdS/ZnS QDs; CdSe/CdS/ZnS QDs interfere with the process of oocyte maturation disfunctioning the cumulus cells or disturbing the signal interaction between germ cell and somatic cell.	[82]
*In vitro*	CdSe/CdS/ZnS QDs	Exposure for 4, 8 and 20 h to 28.9 nmol/L of QDs	Immature oocytes were collected from Kunming mice	QDs enter cumulus cells and accumulate with co-culture time; After being treated for 20 h and being rejected by oocytes, QDs decrease the ratio of oocyte *in vitro* maturation dramatically.	[83]
*In vitro*	CdTe/ZnTe QD-Transferrin bioconjugates	Preantral follicles were treated with escalating concentrations of (0.0289, 0.289, 2.89 and 28.9 nmol/L) CdTe/ZnTe QD-Transferrin bioconjugates for 8 days	Pre antral follicles were collected from female Kunming mice	Dose-dependent up-take of QDs-Tf by theca and granulosa cells; A significant decrease in the rate of antrum cavity formation and the ratio of oocytes with first polar body was observed.	[84]
*In vivo*	TiO_2_-NPs (anatase; NPs were found to aggregate in culture medium and consequently the mean sizes were 240–280 nm (0.1 mg/L) and 259–360 nm (1.0 mg/L)	Reproductively active fish were exposed for 13 weeks to 0.1 and 1.0 mg/L	Zebrafish *Danio rerio*	Chronic exposure to 0.1 mg/L of TiO_2_-NPs can significantly impair zebrafish reproduction; Reduced expression of genes coding for growth factors implicated as paracrine stimuli in the oocyte maturation.	[85]
*In vivo*	TiO_2_-NPs (anatase, average particle size: 6 nm, surface area: 174.8 m^2^/g)	TiO_2_-NPs suspensions at different concentrations (2.5, 5, and 10 mg/kg of body weight) were administered to mice by intragastric administration for 90 consecutive days.	CD-1 (ICR) female mice	TiO_2_-NPs can accumulate in the ovary and result in ovarian damage, causing an imbalance of mineral element distribution, decreased fertility or pregnancy rate and oxidative stress in mice.	[86]
*In vivo*	Ag-NPs (3 and 35 nm)	Exposure to 10 μg/L of Ag-NPs for 35 days	Sheepshead minnows (*Cyprinodon Variegatus*)	Gene expression analysis revealed a dramatic transcriptional response indicating that exposure to these NPs has the potential to cause reproductive dysfunction; Absence of ovary morphological and developmental alterations.	[87]
*In vivo*	TiO_2_-NPs (anatase-75% rutile-25%, average primary particle size: 21 nm, specific surface area: 50 ± 15 m^2^/g)	Reproductively active fish were exposed for 14 days to 0.1 and 1.0 mg/L	Zebrafish *Danio rerio*	Female gonad showed a normal spread of oocyte development stages; The highest TiO_2_-NPs dose caused a markedly lower production of eggs and viable embryos.	[88]
**Disruption of normal levels of sex hormones**					
*In vitro*	Au-NPs (10 nm)	Exposure for 1,3,5 and 24 h to 2.85 × 10^10^ NPs/mL	Rat granulosa cells	Au-NPs induced a greater output of estradiol at 1–5 h of treatment; A significant decrease in estradiol was observed at 24 h.	[89]
*In vitro*	Calcium phosphate-NPs (20–30 nm, less than 10% particles were greater than 100 nm)	Cells were divided into different groups of exposure: control (treated with 4-androstene-3,17-dione), group II (treated with 10 μM of Calcium phosphate-NPs and 4-androstene-3, 17-dione) and group III (treated with 100 μM of Calcium phosphate-NPs and 4-androstene-3, 17-dione) for 48 h	Granulosa cells collected from infertile women	Treatment with calcium phosphate-NPs did not significantly change either the progesterone or estradiol levels in culture fluid; No changes in the expression levels of mRNAs encoding P450scc, P450arom and StAR.	[74]
*In vitro*	CdTe/ZnTe QD-Transferrin bioconjugates	Preantral follicles were treated with escalating concentrations of (0.0289, 0.289, 2.89 and 28.9 nmol/L) CdTe/ZnTe QD-Transferrin bioconjugates for 8 days	Pre antral follicles were collected from female Kunming mice	Increase in 17 β-estradiol secretion, at the 4th-8th day post-treatment, at the highest QD-Tf concentration; 17 β-estradiol secretion decreased compared to controls at lower QD-Tf concentrations.	[84]
*In vivo*	TiO_2_-NPs (anatase, average particle size: 6 nm, surface area: 174.8 m^2^/g)	TiO_2_-NP suspensions at different concentrations (2.5, 5, and 10 mg/kg of body weight) were administered to mice by intragastric administration for 90 consecutive days.	CD-1 (ICR) female mice	Higher serum levels of estradiol; Lower serum levels of progesterone, FSH, LH and testosterone.	[86]
*In vivo*	NRDE-NPs (22–27 nm; particle composition showed a higher percentage of organic carbon than elemental carbon)	Inhalation exposure, for 5 h daily from day 1 to 19 of gestation, to 148.86 μg/m^3^	Pregnant Fischer 344 rats	Increase in LH levels; Decrease in progesterone levels; No significant differences were observed in FSH, prolactin, ir-inhibin and testosterone levels.	[90]
*In vivo*	ZnO-NPs (20–30 nm)	Oral administration of 333.33 mg/kg of ZnO-NPs	Wistar rats	The sexual hormone level (FSH, LH and estradiol) showed no significant difference between control and treatment group.	[91]

8-OHdG, 8-hydroxy-2-deoxyguanosine; Ag-NPs, Silver Nanoparticles; Al_2_O_3_-NPs, Aluminium oxide Nanoparticles; Au-NPs, Gold Nanoparticles; CdS-QDs, Cadmium Sulfide Quantum Dots; CdSe-QDs, Cadmium Selenium core Quantum Dots; CdTe/ZnTe QD, Cadmium Tellurium/Zinc Tellurium Quantum Dots; COX-2, Cyclooxygenase-2; FSH, Follicle Stimulating Hormone; LH, Luteinizing Hormone; MAPKs, Mitogen-Activated Protein Kinases; MN, Micro Nuclei; MARCO, Macrophage Receptor with Collagenous structure; MWCNTs, Multi Walled Carbon Nanotubes; MTT, 3-(4,5-dimethyl-2-thiazol)-2,5-diphenyl-2H-tetrazolium bromide; NRDE-NPs, Nanoparticle-Rich Diesel Exhaust Nanoparticles; P450arom, Aromatase P450; P450scc, Cytochrome P450 side-chain cleavage; ROS, Reactive Oxygen Species; SCE, Sister Chromatid Exchanges; SiCNWs, Silicon Carbide Nanowires; StAR, Steroidogenic Acute Regulatory protein; TiO_2_, Titanium Dioxide; TiO_2_-NPs, Titanium Dioxide nanoparticles; UV, Ultra Violet; ZnO-NPs, Zinc Oxide Nanoparticles; ZnS, Zinc Sulfide; ZnS-QDs, Zinc Sulfide Quantum Dots.

**Table 4:** Estrogenic effects of NPs.

Type of study	Type and physico-chemical properties of NPs	Experimental protocol	Cell line/laboratory animals	Results	References
*In vitro*	CdTe-QDs (~3 nm)	Treatment with 0.5 and 10 μg/mL for time periods ranging from 5 min to 96 h	Human breast cancer MCF-7 cells	CdTe-QDs induced estrogen-related genomic and non-genomic signaling pathway; Increased estrogen receptor- α activation and biphasic phosphorylation of AKT and ERK1/2.	[99]
*In vivo*	CdS-QDs (4.2 ± 1 nm)	Exposure for 21 days to 5, 50 and 500 μg/L of CdS-QDs	Male sticklebacks (*Gasterosteus aculeatus*)	Induction of VTG synthesis was not detected in any of the treatment groups.	[95]
*In vivo*	NRDE-NPs—Averages of mode diameter and number concentrations for low, middle and high doses were 22.48, 26.13 and 27.06 nm; 2.27 × 10^5^, 5.11 × 10^5^ and 1.36 × 10^6^, respectively	Inhalation exposure to low (15.37), middle (36.35) and high (168.84) μg/m^3^ doses for 4, 8, or 12 weeks (5 hours/day, 5 days/week)	Fischer F344 rats	Higher plasma progesterone levels after exposure for 4 weeks to middle or high NRDE-NPs concentrations; Lower plasma progesterone levels in rats treated with low or high NRDE-NPs after 8 weeks.	[33]
*In vivo*	NRDE-NPs—Averages of mode diameter and number concentrations were 26.81 nm and 1.83 × 10^6^, respectively	Inhalation exposure to 148.86 μg/m^3^ for 5 h daily for 19 gestational days	Pregnant Fischer F344 rats	Significantly decreased serum progesterone concentrations in male offspring.	[34]
*In vivo*	(CdS)/CdTe capped-QDs	Exposure for 48 h to increasing concentrations of (CdS)/CdTe capped-QDs (1, 2 and 6 μg/L)	Juvenile rainbow trout (*Oncorhynchus mykiss*)	Up-regulation of VTG; Down-regulation of VTG receptor.	[94]
*In vivo*	C_60_ fullerene	Male zebrafish (Danio rerio) were fed for 5 days with brine shrimp preparations that had accumulated a mixture of C_60_ fullerene (10% *v*/*v* of the 600 mg C60/900 mL water) and 1 μg/L of 17α-ethinylestradiol or C_60_ fullerene or 17α-ethinylestradiol alone.	Male zebrafish (*Danio rerio*)	The treatment with C_60_ fullerene reduced the biodisponibility of synthetic estrogen, 17α-ethinylestradiol.	[97]
*In vivo*	CB-NPs (50–60 nm)	Intratracheal instillation at a total dose of 11, 54 and 268 μg/animal during gestation (7, 10, 15 and 18 gestation days)	C57BL/6 mice	Exposure to 11 μg/animal induced a significantly earlier onset of puberty, assessed as time of vaginal opening.	[100]
*In vivo*	C_60_ fullerene	Exposure of male zebrafish (*Danio rerio*) to increasing concentrations of C_60_ fullerene. See Park *et al*. (2010)	Male zebrafish (*Danio rerio*)	Bioavailability of 17α-ethinylestradiol was reduced with increasing concentration of C_60_ fullerene and was related to computed surface area of C_60_ fullerene.	[98]
*In vivo*	Ag-NPs (20 nm)	Exposure to increasing concentrations of Ag-NPs (0.06, 0.6 and 6 μg/L) for 96 h	Juvenile rainbow trout (*Oncorhynchus mykiss*)	Ag-NPs induced a significant decrease in liver expression of VTG-like proteins.	[93]
*In vivo*	NRDE-NPs Low dose group (average of mode diameter: 22.78 ± 0.39 nm; mass concentration: 41.73 ± 0.58 μg/m^3^; number concentration: 8.21 × 10^5^ ± 3.1 × 10^5^), High dose group (average of mode diameter: 26.31 ± 0.38 nm; mass concentration: 152.01 ± 1.18 μg/m^3^; number concentration: 1.8 × 10^6^ ± 5.18 × 10^5^)	Exposure for 8 weeks (5 h/day, 5 days/week) to 41.73 μg/m^3^ and 152.01 μg/m^3^ of NRDE-NPs	Male C57BL/Jcl mice	Corticosterone and progesterone concentrations increased significantly in mice exposed to low concentration; Corticosterone and progesterone concentrations decreased significantly in mice exposed to high-concentration.	[96]
*In vivo*	Ag-NPs (23.5 ± 4.4 nm)	Exposure to 1 and 25 μg/L of Ag-NPs for 28 days	Male Medaka (*Oryzias latipes*) fish	Conspicuous mRNA induction of VTG-1 and Chg-L in livers of exposed fish.	[92]

Ag-NPs, Silver Nanoparticles; AKT, serine/threonine-protein kinases; CB-NPs, Carbon Black Nanoparticles; CdS-QDs, Cadmium Sulfide Quantum Dots; CdS/CdTe capped-QDs, Cadmium Sulfide/Cadmium Tellurium capped-Quantum Dots; CdTe-QDs, Cadmium Tellurium Quantum Dots; Chg-L, choriogenin L; ERK, extracellular signal-regulated kinase; NRDE-NPs, Nanoparticle-Rich Diesel Exhaust Nanoparticles; VTG, Vitellogenin.

**Table 5:** Other effects of NPs on endocrine system.

Type of study	Type and physico-chemical properties of NPs	Experimental protocol	Cell line/laboratory animals	Results	References
**Effects on thyroid function**					
*In vitro*	Ag-NPs (2–6 and 10 nm), ZnO-NPs (2–10 and 9 nm) and QDs composed of cadmium telluride (2–10 and 10–15 nm)	Exposure of cultured tail fin biopsy to 0.06 μg/L–5.5 mg/L of Ag-NPs, 0.19–10 mg/L of ZnO-NPs and 0.25 μg/L–22 mg/L of QDs for 48 h	Cultured tail fin biopsy assay derived from *Rana catesbeiana* tadpoles	Reduced levels of transcripts encoding TRβ and RLKI were induced by exposure to 5–10 nM of Ag-NPs and to 0.1 nM of QDs; Treatment with ZnO-NPs had no effect on TRβ and RLKI transcript levels; Ag-NPs were able to affect the TH-action on TRβ and RLKI transcripts according to an “inverted-U” hormesis response.	[105]
*In vivo*	Cr-NPs (40–70 nm)	Oral administration of 150, 300 and 450 μg/Kg of Cr-NPs for 8 weeks	Male Sprague Dawley rats	Cr-NPs did not result in significant modifications in TSH, FT_3_ and FT_4_ serum levels.	[106]
**Effects on insulin action and metabolism**					
*In vitro*	TiO_2_-NPs (21 nm)	Exposure for 2 h to 50 and 200 μg/mL of TiO_2_-NPs	Fao rat hepatoma cells	TiO_2_-NPs impaired insulin response and induced insulin resistance.	[110]
*In vitro*	CeO_2_-NPs (100 nm)	Exposure to 100 nmol/L of CeO_2_-NPs, alone or in combination with 30 nmol/L of sodium selenite for 1–6 days	Pancreatic islets	Increased cell viability, ATP/ADP ratio and secretion of insulin in response to glucose stimuli.	[111]
*In vivo*	CrCl_3_-NPs (40–70 nm)	Dietary supplementation for 35 days with 200 μg/kg of CrCl_3_-NPs	Crossbred pigs (Duroc X Landrace X Yorkshire)	The treatment with CrCl_3_-NPs reduced serum insulin and cortisol levels and increased serum insulin-like growth factor.	[112]
*In vivo*	Cr-NPs (40–50 nm)	Oral administration for 6 weeks of 75, 150, 300, 450, 600 and 1200 ppb of Cr-NPs	Male Sprague Dawley rats	Addition of 300 and 450 ppb of Cr-NPs reduced the serum insulin level.	[113]
*In vivo*	Cr-NPs (40–70 nm)	Oral administration of 150, 300 and 450 μg/Kg of Cr-NPs for 8 weeks	Male Sprague Dawley rats	Dietary supplementation of 150, 300, and 450 μg/kg significantly decreased serum concentrations of insulin and cortisol and increased sera levels of IGF-I.	[106]
*In vivo*	Double walled-CNTs (0.5–2.5 nm for inner tubes and 1.2–3.2 nm for outer tubes)	Intranasal instillation of 1.5 mg/kg	Male Swiss mice	Increased serum levels of IGF-1.	[114]
*In vivo*	CeO_2_-NPs	Intraperitoneal injection of 60 mg/Kg of CeO_2_-NPs for 2 weeks, alone or in combination with sodium selenite (5 μmol/kg/day)	Male Wistar rats	Improvement in biomarkers of diabetes (oxidative stress, ADP/ATP, glycemic and lipid profile).	[115]
**Effects on neuroendocrine system**					
*In vitro*	C_60_ fullerene	Exposure for 4 h to 100 μM of C_60_ fullerene	Adrenal chromaffin cells obtained from Wistar rats	Treatment with C_60_ fullerene notably increased cell survival and prevented cell death, including apoptosis.	[116]
*In vitro*	MnO-NPs (40 nm) and Ag-NPs (15 nm)	Exposure for 24 h to increasing concentrations (1–100 μg/mL ) of NPs	PC-12 cells derived from *Rattus norvegicus* pheochromocytoma (CRL-1721)	MnO-NP dose dependently depleted DA and DOPAC; Ag-NPs significantly reduced DA and DOPAC at concentrations of 50 μg/mL; HVA levels decreased at 50 μg/mL in both MnO-NP and Ag-NP exposure.	[117]
*In vitro*	Cu-NPs (90.9 ± 19.3 nm), Mn-NPs (52.1 ± 23.8 nm) and Ag-NPs (18.3 ± 7.3 nm)	Treatment for 24 h with 10 μg/mL of NPs and with increasing concentrations of Cu-NPs (2.5, 5, 7.5, 10 and 25 μg/mL)	PC-12 cells	Cu-NPs induced a significant reduction in the content of DA, DOPAC, and HVA in PC12 cells.	[118]
*In vitro*	Au-NPs (28 nm) and Ag-NPs (61 nm)	Treatment for 24 and 48 h with 0.01–1 nM of NPs	Primary culture murine adrenal medullary chormaffin cells harvested from wild-type brown male mice (C57BL/6J)	Exposure to Au-NPs and Ag-NPs caused lower and slower secretion of epinephrine molecules.	[119]
*In vitro*	Carboxyl QDs with CdSe core and ZnS shell (7–8 nm)	Exposure for 24 h to increasing concentrations of QDs (5, 8, 16 and 36 nM)	Mouse chromaffin cells obtained from young C57BL/6J male mice	Exposure to carboxyl QDs with CdSe core and ZnS shell impairs Ca^2+^ influx and severely interferes with the functionality of the exocytotic machinery, compromising the overall catecholamine supply from chromaffin cells.	[120]
*In vitro*	Ag-NPs coated with citrate (6 nm) or polyvinylpyrrolidone (21 nm)	Exposure for 24 h to different concentrations (1–30 μM) of Ag-NPs	PC-12 cells	Treatment with citrate- coated Ag-NPs reduced the emergence of the acetylcholine-phenotype; Treatment with polyvinylpyrrolidone -coated Ag-NPs enhanced differentiation into a DA-phenotype.	[121]
*In vitro*	Silica NPs (15 nm)	Exposure for 24 h to 25–200 μg/mL of silica NPs	PC-12 cells	Silica NPs exerted a dose-dependent reduction in DA synthesis related to redundant ROS generation in the cells.	[122]
*In vitro*	Citrated-capped Ag-NPs (from 15 to 60 nm); PEG (25.6 ± 7.8 nm) and heparin (20.6 ± 15.3 nm) surface modified Au NPs.	Exposure for 24 h to citrated-capped Ag-NPs at 1 nM concentration and to PEG and heparin Au NPs at 10 μg/mL	Primary culture murine adrenal medullary chormaffin cells harvested from wild-type brown male mice (C57BL/6J)	Noble metal NPs disrupt exocytosis, typically altering the number of molecules and kinetics of release.	[123]
**Effects on pituitary gland**					
*In vivo*	CrCl_3_-NPs (40–70 nm)	Dietary supplementation for 35 days with 200 μg/kg of CrCl_3_-NPs	Crossbred pigs (Duroc X Landrace X Yorkshire)	No significant difference in GH level between exposed and control group.	[112]
*In vivo*	CrCl_3_-NPs (40–70 nm)	Dietary supplementation for 35 days with 200 μg/kg of CrCl_3_-NPs	Crossbred pigs (Duroc X Landrace X Yorkshire)	CrCl_3_-NPs significantly increased the mean level, lowest value, peak value and peak duration of GH; Pituitary mRNA expression of GH improved significantly.	[124]
*In vivo*	Cr-NPs (40–70 nm)	Oral administration of 150, 300 and 450 μg/Kg of Cr-NPs for 8 weeks	Male Sprague Dawley rats	No significant alterations in the concentrations of pituitary produced GH.	[106]
**Effects on adrenal gland**					
*In vivo*	NRDE-NPs—Averages of mode diameter and number concentrations for low, middle and high doses were 22.48, 26.13 and 27.06 nm; 2.27 × 10^5^, 5.11 × 10^5^ and 1.36 × 10^6^, respectively	Inhalation exposure to low (15.37), middle (36.35) and high (168.84) μg/m^3^ doses for 4, 8, or 12 weeks (5 h/day, 5 days/week)	Fischer F344 rats	No significant differences in plasma concentrations of corticosterone were observed in the different exposure groups.	[33]
*In vivo*	NRDE-NPs—Averages of mode diameter and number concentrations were 26.81 nm and 1.83 × 10^6^, respectively	Inhalation exposure to 148.86 μg/m^3^for 5 h daily for 19 gestational days	Pregnant Fischer F344 rats	Significantly lower corticosterone concentrations were observed in male offspring.	[34]
*In vivo*	Cr-NPs (40–70 nm)	Oral administration of 150, 300 and 450 μg/Kg of Cr-NPs for 8 weeks	Male Sprague Dawley rats	Exposure to Cr-NPs decreased plasma cortisol levels.	[106]
*In vivo*	NRDE-NPs Low dose group (average of mode diameter: 22.78 ± 0.39 nm; mass concentration: 41.73 ± 0.58 μg/m^3^; number concentration: 8.21 × 10^5^ ± 3.1 × 10^5^), High dose group (average of mode diameter: 26.31 ± 0.38 nm; mass concentration: 152.01 ± 1.18 μg/m^3^; number concentration: 1.8 × 10^6^ ± 5.18 × 10^5^)	Exposure for 8 weeks (5 h/day, 5 days/week) to 41.73 μg/m^3^ and 152.01 μg/m^3^ of NRDE-NPs	Male C57BL/Jcl mice	At the highest exposure dose, NRDE-NPs inhibited basal and ACTH-stimulated corticosterone release; The lowest NRDE-NP concentration increased synthesis and release of corticosterone.	[96]
*In vivo*	NRDE-NPs (22–27 nm; particle composition showed a higher percentage of organic carbon than elemental carbon)	Inhalation exposure to 148.86 μg/m^3^ for 5 h daily from day 1 to 19 of gestation	Pregnant Fischer 344 rats	Increased serum levels of corticosterone.	[90]

ACTH, AdrenoCorticoTropic Hormone; ADP, Adenosine Diphosphate; ATP, Adenosine Triphosphate; Ag-NPs, Silver Nanoparticles; Au-NPs, Gold Nanoparticles; CdSe, Cadmium Selenium; CeO_2_-NPs, Cerium Oxide Nanoparticles; CNTs, Carbon Nanotubes; Cr-NPs, Chromium Nanoparticles; CrCl_3_-NPs, Chromium Chloride Nanoparticles; Cu-NPs, Copper nanoparticles; DA, Dopamine; DOPAC, dihydroxyphenylacetic acid; FT_3_, free triiodothyronine; FT_4_, free thyroxine; GH, Growth Hormone; HVA, HomoVanillic Acid; IGF-I, Insulin-like Growth Factor I; Mn-NPs, Manganese Nanoparticles; MnO-NPs, Manganese Oxide Nanoparticles; NRDE-NPs, Nanoparticle-Rich Diesel Exhaust Nanoparticles; PEG, PolyEthylene Glycol; QDs, Quantum Dots; RLKI, Rana Larval Keratin Type I; ROS, Reactive Oxygen Species; TiO_2_-NPs, Titanium Dioxide nanoparticles; TRβ, Thyroid hormone Receptor β; TSH, Thyrotropic-Stimulating Hormone; ZnO-NPs, Zinc Oxide Nanoparticles; ZnS, Zinc Sulfide.

## Abbreviations

8-OHdG: 8-hydroxy-2-deoxyguanosine
ACTH: AdrenoCorticoTropic Hormone
ADP: Adenosine Diphosphate
Ag-HC-NPs: Hydrocarbon-coated silver Nanoparticles
Ag-NPs: Silver Nanoparticles
Ag-PS-NPs: Polysaccharide-coated silver Nanoparticles
Al-NPs: Aluminum Nanoparticles
Al_2_O_3_-NPs: Aluminium Oxide Nanoparticles
ATP: Adenosine Triphosphate
Au-NPs: Gold Nanoparticles
C_60_HyFn: hydrated C_60_ fullerene
CB: Carbon Black
CB-NPs: Carbon Black Nanoparticles
CdS-QDs: Cadmium Sulfide Quantum Dots
CdS/CdTe capped-QDs: Cadmium Sulfide/Cadmium Tellurium capped-Quantum Dots
CdSe-core-QDs: Cadmium Selenium core Quantum Dots
CdTe-QDs: Cadmium Tellurium Quantum Dots
CdTe/ZnTe QDs: Cadmium Tellurium/Zinc Tellurium Quantum Dots
CeO_2_-NPs: Cerium Oxide Nanoparticles
Chg: Choriogenin
CHO: Chinese Hamster Ovary cells
CNTs: Carbon Nanotubes
COX-2: Cyclooxygenase-2
CrCl_3_-NPs: Chromium Chloride Nanoparticles
Cr-NPs: Chromium Nanoparticles
Cu-NPs: Copper nanoparticles
DA: Dopamine
DE-NPs: Diesel Exhaust Nanoparticles
DOPAC: dihydroxyphenylacetic acid
DSP: Daily Sperm Production
EDCs: Endocrine Disrupting Chemicals
EDPL: Endocrine Disruptor Priority List
ER: Estrogenic Receptor
ERK: extracellular signal-regulated kinase
EuOH_3_-NPs: Europium Hydroxied Nanoparticles
Eu_2_O_3_-NPs: Europium Oxide Nanoparticles
Fe_3_O_4_-NPs: Magnetic Iron Oxide Nanoparticles
FSH: Follicle Stimulating Hormone
FT_3_: free triiodothyronine
FT_4_: free thyroxine
GH: Growth Hormone
GnRH: Gonadotropin Releasing Hormone
HMG-CoA: 3-hydroxy-3-methylglutaryl-coenzyme A
HVA: HomoVanillic Acid
IGF-I: Insulin-like Growth Factor I
IR: Insulin Resistance
LDL-R: Low Density Lipoprotein Receptor
LH: Luteinizing Hormone
MAPKs: Mitogen-Activated Protein Kinases
MARCO: Macrophage Receptor with Collagenous structure
MN: Micro Nuclei
MnO-NPs: Manganese Oxide Nanoparticles
MoO_3_-NPs: Molybdenum Trioxide Nanoparticles
mPEG@Au-NP: ω-methoxy poly(ethylene glycol) capped gold-NPs
MTT: 3-(4,5-dimethyl-2-thiazol)-2,5-diphenyl- 2H-tetrazolium bromide
MWCNTs: Multi Walled Carbon Nanotubes
NPs: NanoParticles
NRDE-NPs: Nanoparticle-Rich Diesel Exhaust Nanoparticles
nSP: amorphous nanosilica particles
Ntera2, NT2: human testicular embryonic carcinoma cell line
P450 17α: Cytochrome P450 17α
P450 17β-HSD: Cytochrome P450 17β-HydroxySteroid Dehydrogenase
P450arom: Aromatase P450
P450scc: Cytochrome P450 side-chain cleavage
PBR: Peripheral-type Benzodiazepine Receptor
PEG: PolyEthilen-Glycol
PEG-NH2@Au-NP: ω-aminoethyl poly(ethylene glycol) capped gold-NPs
POP: Persistent Organic Pollutants
QDs: Quantum Dots
RLKI: Rana Larval Keratin Type I
ROS: Reactive Oxygen Species
SCE: Sister Chromatid Exchanges
SiCNWs: Silicon Carbide Nanowires
s-MWCNTs-PEG: synthesized functionalized MWCNTs with polyethylene glycol
SR-B1: Scavenger Receptor class B type 1
SSC: Spermatogonial Stem Cells
StAR: Steroidogenic Acute Regulatory protein
T2DM: Type 2 Diabetes Mellitus
TH: Thyroid Hormone
TiO_2_: Titanium Dioxide
TiO_2_-NPs: Titanium Dioxide nanoparticles
TRβ: Thyroid hormone Receptor β
TSH: Thyrotropic-Stimulating Hormone
UV: Ultra Violet
VTG: Vitellogenin
ZnO-NPs: Zinc Oxide Nanoparticles
ZnS: Zinc Sulfide
ZnS-QDs: Zinc Sulfide Quantum Dots
